# Amorphous and Polycrystalline Photoconductors for Direct Conversion Flat Panel X-Ray Image Sensors

**DOI:** 10.3390/s110505112

**Published:** 2011-05-09

**Authors:** Safa Kasap, Joel B. Frey, George Belev, Olivier Tousignant, Habib Mani, Jonathan Greenspan, Luc Laperriere, Oleksandr Bubon, Alla Reznik, Giovanni DeCrescenzo, Karim S. Karim, John A. Rowlands

**Affiliations:** 1 Department of Electrical and Computer Engineering, University of Saskatchewan, Saskatoon, SK, S7N 5A9, Canada; E-Mails: joel.frey@usask.ca (J.B.F.); george.belev@lightsource.ca (G.B.); 2 Anrad Corporation, 4950 rue Lévy, Saint-Laurent, QC, H4R 2P1, Canada; E-Mails: tousignanto@anrad.com (O.T.); manih@anrad.com (H.M.); greenspj@anrad.com (J.G.); laperrierel@anrad.com (L.L.); 3 Department of Physics, Lakehead University, 955 Oliver Road, Thunder Bay, ON, P7B 5E1, Canada; E-Mails: obubon@lakeheadu.ca (O.B.); reznika@tbh.net (A.R.); 4 Thunder Bay Regional Research Institute, 980 Oliver Road, Thunder Bay, ON, P7B 6V4, Canada; E-Mails: decrescg@tbh.net (G.D.); rowlandj@tbh.net (J.A.R.); 5 Department of Electrical and Computer Engineering, University of Waterloo, Waterloo, ON, N2L 3G1, Canada; E-Mail: kkarim@uwaterloo.ca; 6 Imaging Research, Sunnybrook Health Sciences Centre, University of Toronto, 2075 Bayview Avenue, Toronto, ON, M4N 3M5, Canada

**Keywords:** x-ray image sensor, detector, direct conversion, x-ray photoconductor

## Abstract

In the last ten to fifteen years there has been much research in using amorphous and polycrystalline semiconductors as x-ray photoconductors in various x-ray image sensor applications, most notably in flat panel x-ray imagers (FPXIs). We first outline the essential requirements for an ideal large area photoconductor for use in a FPXI, and discuss how some of the current amorphous and polycrystalline semiconductors fulfill these requirements. At present, only stabilized amorphous selenium (doped and alloyed a-Se) has been commercialized, and FPXIs based on a-Se are particularly suitable for mammography, operating at the ideal limit of high detective quantum efficiency (DQE). Further, these FPXIs can also be used in real-time, and have already been used in such applications as tomosynthesis. We discuss some of the important attributes of amorphous and polycrystalline x-ray photoconductors such as their large area deposition ability, charge collection efficiency, x-ray sensitivity, DQE, modulation transfer function (MTF) and the importance of the dark current. We show the importance of charge trapping in limiting not only the sensitivity but also the resolution of these detectors. Limitations on the maximum acceptable dark current and the corresponding charge collection efficiency jointly impose a practical constraint that many photoconductors fail to satisfy. We discuss the case of a-Se in which the dark current was brought down by three orders of magnitude by the use of special blocking layers to satisfy the dark current constraint. There are also a number of polycrystalline photoconductors, HgI_2_ and PbO being good examples, that show potential for commercialization in the same way that multilayer stabilized a-Se x-ray photoconductors were developed for commercial applications. We highlight the unique nature of avalanche multiplication in a-Se and how it has led to the development of the commercial HARP video-tube. An all solid state version of the HARP has been recently demonstrated with excellent avalanche gains; the latter is expected to lead to a number of novel imaging device applications that would be quantum noise limited. While passive pixel sensors use one TFT (thin film transistor) as a switch at the pixel, active pixel sensors (APSs) have two or more transistors and provide gain at the pixel level. The advantages of APS based x-ray imagers are also discussed with examples.

## 1. Introduction: Direct Conversion Flat Panel X-Ray Imagers

Flat panel x-ray imagers (FPXIs) are now widely used in digital x-ray imaging with applications in medical, security and industrial imaging. Such flat panel x-ray image sensors, also called x-ray image detectors, have to be large area due to the lack of a practical means to focus x-rays which necessitates a shadow x-ray image which is larger than the object (e.g., the body part) to be imaged. Their most important applications are in medical imaging such as mammography, chest radiology, angiography, fluoroscopy, computed tomography, and offer a number of distinct advantages over other types of digital sensors (e.g., [1–10]). Further, digital flat-panel detectors make it possible to view combined x-ray and magnetic resonance images to more-accurately guide medical diagnosis and treatment [11]. There are essentially two types of FPXIs based on the technique used to detect the x-rays [1,12]. In *indirect conversion* based FPXIs, the x-rays are first converted to light via a scintillating phosphor, such as CsI:Tl (which absorbs the incident x-rays), and then the light emitted from the scintillator is detected by an array of photodiodes; a recent example may be found in [13]. In *direct conversion* FPXIs, an x-ray photoconductor is used as the principal detecting element to convert the absorbed x-ray photons directly to collectable charge carries, which represent the signal. There have been several reviews on this topic: see, for example, [14–19]. In this review we concentrate only on direct conversion FPXIs, and highlight some of the new advances in the field and the progress made to date with special mention of the work done in Canada, given the purpose of the special issue—the state of the art in Canada.

**Figure 1.**
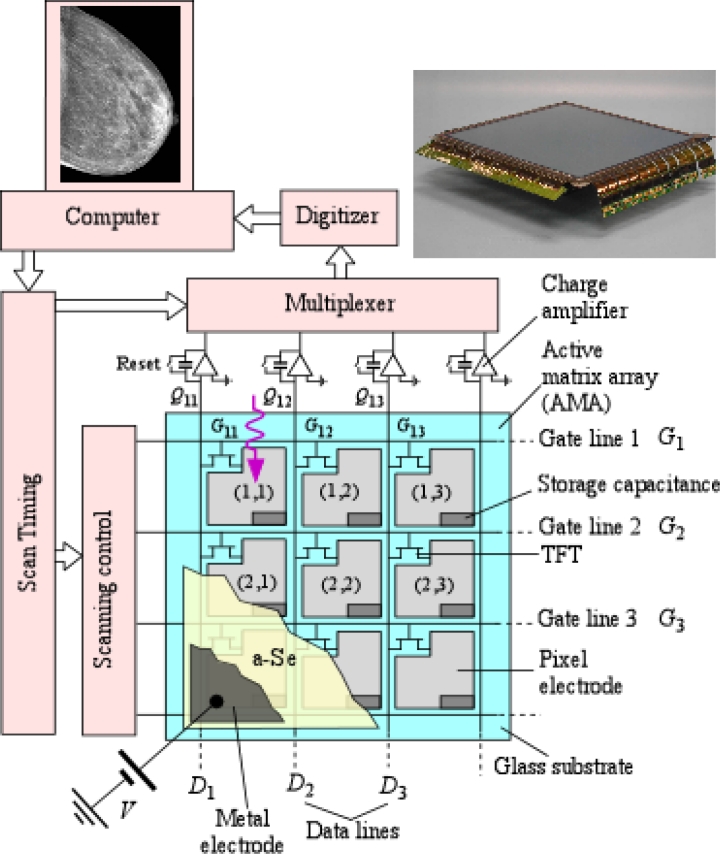
A simplified schematic illustration of a FPXI and its peripheral electronics that drive the sensor operation. At its base, the FPXI has a TFT-AMA with *M* × *N* number of pixels (e.g., 2,816 × 3,584) as a substrate. There is an x-ray photoconductor (a-Se) deposited on the AMA, and a top electrode to apply a voltage to the photoconductor. The x-rays absorbed in the a-Se layer above pixel (1,1) generate charges that drift and become collected and stored on the storage capacitor *C*_11_ at this pixel. If a signal is applied to the gate *G*_11_ of the TFT at pixel (1,1), by activating the gate line *G*_1_, it conducts (it switches on) and the charge *Q*_11_ on *C*_11_ is transferred to the data line and hence to the external electronics. Data lines feed into charge amplifiers. The *C*_11_ and TFT structures are not inside the glass substrate but on the surface of the glass substrate as indicated in Figure 2. The activation of the gate line *G*_1_ allows the charges *Q*_11_, *Q*_12_, *Q*_13_ *etc.* to be read at the same time to a multiplexer and then onto a digitizer *etc*. At the end the read-cycle for the 1st row, the 2nd row is addressed via *G*_2_ and so on until all the rows are sequentially addressed, and hence the whole image is read out.

A flat panel x-ray imager consists of a large array of pixels as part of an active matrix array (AMA) as illustrated in Figure 1. An AMA is a two dimensional array of pixels in which each pixel has a thin film transistor (TFT) that can be externally addressed. The TFT AMA technology was pioneered by Peter Brody using CdSe TFTs in early 1970s [20]. As shown in the figure, each pixel is identical with its TFT gate connected to a particular address line and the source to a particular data line. The AMA has *M* × *N* number of gate and data lines (Figure 1) in which *M* and *N* can be very large e.g., 2,816 × 3,584 in the sensor shown in Figure 1.

**Figure 2.**
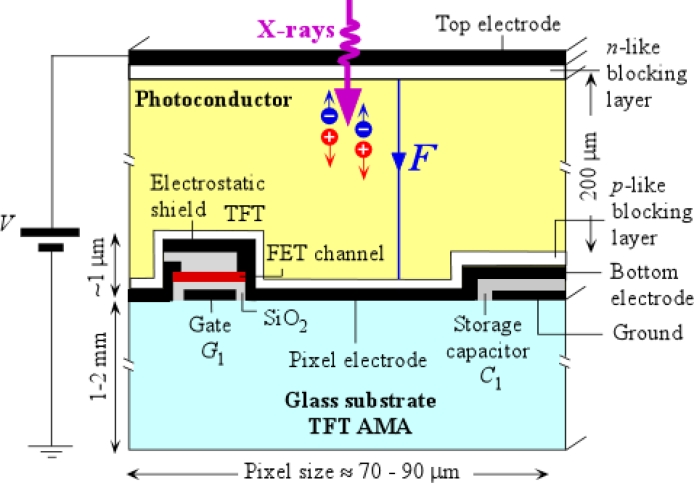
A simplified schematic diagram of the cross section of a single pixel with a TFT. The charges generated by the absorption of x-rays drift towards their respective electrodes. The capacitor *C*_1_ integrates the induced current due to the drift of the carriers which results in a stored charge *Q*_1_ on *C*_1_. The TFT is normally off and is turned on when the gate *G*_1_ is addressed. (Not to scale).

The active matrix array is coated by a suitable x-ray photoconductor material, such as stabilized amorphous selenium (a-Se), which is then electroded on its surface to allow the application of a bias voltage. Thus, each pixel acts as an individual x-ray detector and has a biased photoconductor as illustrated in the schematic cross section of a pixel depicted in Figure 2. There is a storage capacitor at each pixel to collect charges that are generated by the photoconductor. The applied bias voltage establishes an electric field inside the photoconductor so that the charge carriers released by the absorption of an x-ray photon can be drifted and “collected” in the sense that they result in the deposition of charge on *C*_1_. As mentioned later, *C*_1_ actually integrates the induced current by the drift of the carriers; and the integrated current, the charge on *C*_1_, represents what appears to be collected from the photogenerated carriers. In the example shown in Figure 2, Pixel 1 receives the radiation, and the photogenerated charge in the photoconductor is collected on the storage capacitor *C*_1_. In Figure 2 we show an a-Si:H (hydrogenated amorphous silicon) TFT switch, which allows the charge *Q*_1_ on *C*_1_ to be read out into the external circuit that has a charge amplifier as indicated in Figure 1. When the gate *G*_1_ of the TFT_1_ is activated, the TFT_1_ switches on, and the charge on *C*_1_ is readout as *AQ*_1_ whre *A* is the amplifier gain. The amount of charge *Q*_1_ that is generated depends on the incident radiation *X*_1_ on that particular pixel inasmuch as the number of electron and hole pairs generated in the photoconductor is proportional to the photon flux and the photon energy. One can therefore represent the x-ray image in terms of the charges residing on the pixel storage capacitors of the FPXI. Prototype a-Se based FPXIs were first demonstrated by Rowlands and coworkers [21–25] and by Lee, Cheung and Jeromin [26,27] in the mid-nineties. Since the first demonstration of the a-Se based FPXI, much research has been done in characterizing and understanding its imaging properties as, for example, reported in references [28–30]. In hind-sight, once TFT-AMAs were developed for the flat panel display industry, it was obvious that it was only a matter of time that either indirect or direct conversion FPXIs would be developed [1,19,31].

It is apparent from Figure 1 that the pixel electrode does not cover the whole pixel area and gives the impression that not all the incident x-rays are utilized. It appears that the regions outside the collection electrode are essentially “dead areas” which are not involved in the conversion of the absorbed x-rays to the pixel signal charge. A fill factor (FF) is used to describe what percentage area of the pixel is actually useful in capturing the incident radiation and converting it to a signal; the fraction that is sensitive to the incident radiation. The geometric FF for the TFT-AMAs used in direct conversion FPXIs depends on the AMA design and the application, and are typically in the range 75–85%. The effective FF, however, is higher, nearly 100% because the field in the dead zone bends towards the pixel electrode and enables photogenerated charges in the deadzone to be collected [1,9,28].

Cost-effective production of commercial FPXIs requires the TFT-AMA panel to have very few defects (which reflect in blurs and distortions in the image), reproducible characteristics, and to be relatively inexpensive and easily obtainable, inasmuch as FPXI manufacturers procure the TFT-AMA panel from third party sources. The importance of the TFT-AMA design in both indirect and direct conversion FPXIs cannot be overstated, and has been recently reviewed by Antonuk [32]. The TFT-AMA cross sections in Figures 1 and 2 show a passive pixel in which the TFT acts only as an addressable switch to transfer the charge accumulated on *C*_1_ to the external circuit. It is possible to introduce one or more additional TFTs at each pixel and introduce gain for the sensor at the pixel level; and hence turn the pixel into an active pixel sensor (APS). Such TFT arrays are called *active-pixel sensor* AMAs and have the potential of providing higher detective quantum efficiency at lower dose [33,34]. More complicated pixel electronics, of course, implies a more complex fabrication process and hence implies a higher cost. On the other hand, there have been a number of advances in this field in the last ten years [33–37], and active pixel FPXIs offer a number of potential advantages that could outweigh the cost-disadvantage in the future.

Figure 2 shows a schematic cross section of a single pixel showing the electronics on the glass substrate. In current commercial direct conversion FPXIs, the photoconductor of choice is stabilized a-Se, which is not used as a simple layer but rather as a multilayer structure. The a-Se photoconductor shown in Figure 2 actually has thin blocking layers between the photoconductor and the contacts to prevent charge injection from the contacts to reduce the dark current. The reasons for choosing a-Se will become apparent below in Section 2 but the reader can also find more information on its historical development in reference [15]. At present there are several manufacturers that have either brought out an a-Se based flat panel x-ray sensor or have been reported to have plans in developing one [38]. Imaging properties of a-Se based FPXIs for various medical imaging modalities have been extensively examined and analyzed, and there are a number of papers with detailed analyses; see for example [30,39–45].

In addition to a-Se, there have been a number of other photoconductors, such as polycrystalline layers of TlBr [46,47], PbI_2_ [48,49], HgI_2_ [50–56], CdZnTe [57,58] and PbO [59,60], that have been investigated and some of these, in particular HgI_2_ and PbO, have shown potential for use in commercial FPXI applications. Most of these photoconductors to date either suffer from possessing too large a dark current or not having sufficient charge collection efficiency. In some cases, there are technological problems in manufacturing a uniform and homogenous layer over a large area. Nonetheless, with dedicated research, these problems are likely to be solved in the same way one had to solve similar problems in the development of commercial a-Se FPXIs.

**Figure 3.**
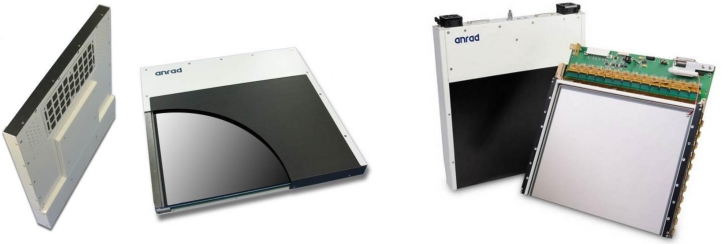
Anrad’s mammographic FPXI AXS-2430 (previously LMAM) is used in the USA and European mammography markets. The field of view is 24 cm × 30 cm. These FPXIs have a pixel pitch of 85 μm, high DQE, high MTF, high contrast, high dynamic range, high patient throughput, and are capable of tomosynthesis.

**Figure 4.**
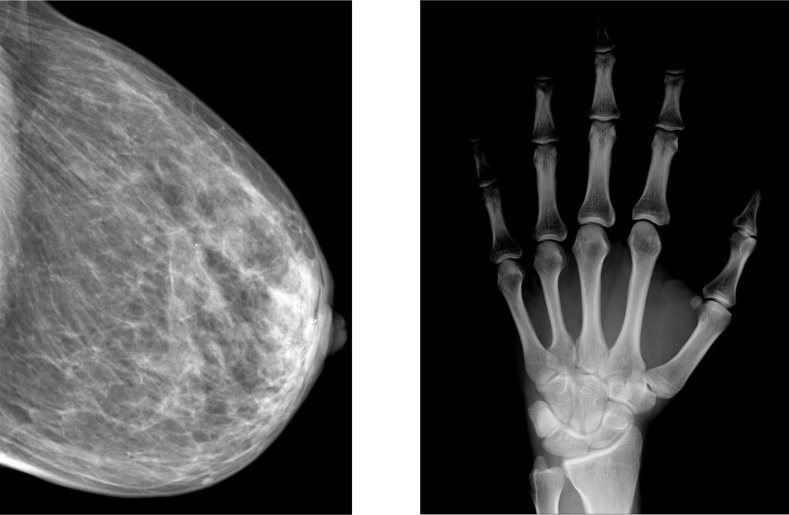
Two typical x-ray images from an a-Se FPXI. Left, a typical x-ray image of a breast. Right, an x-ray image of a hand.

Figure 3 shows a stabilized a-Se based FPXI (AXS-2430) for mammography that has been developed and marketed by Anrad. Figure 4 shows two examples of x-ray images taken by an a-Se FPXI: a breast and a hand. The particular FPXI in Figure 3 has a field of view of 24 cm × 30 cm and the pixel pitch is 85 μm. There are 2,816 × 3,584 pixels in the sensor, each pixel being essentially an x-ray detector that, as mentioned above, generates an amount of charge that is proportional to the incident x-ray dose. Because such sensors can capture and process images in a very short time, they can be used in tomosynthesis (the three dimensional reconstruction of an object using several x-ray images taken at different angles), which is a distinct advantage. For example, the mammographic detector in Figure 3 can acquire up to 3 frames per second in the breast tomosynthesis mode without the need for binning (connecting two or more pixels in parallel to increase the signal). In tomosythesis, the detector captures *N* images at different angular views with the total exposure kept about the same to avoid increasing the patient dose. Each frame therefore has 1/*N* amount of exposure, that is, fewer photons than in conventional mammographic imaging. To make up for the reduced number of photons, the pixels can be binned at the expense of resolution. The choice between binning *vs.* high resolution is obviously quite important, and depends on a number of factors as discussed by Zhao [61]. It should be remarked that the a-Se detector marketed by Hologic has a different structure than that shown in Figure 2; an insulating organic layer is used between the positive electrode and a-Se to block hole injection and reduce the dark current. Hologic’s recently patented detector structure is capable of both static imaging and tomosynthesis [62].

The FPXI shown in Figure 3 has been commercialized for mammography in which the average x-ray photon energy is around 20 keV. Larger area FPXIs e.g., 9 in × 9 in and 14 in × 14 in, with a thicker a-Se photoconductor for use in general radiology and fluoroscopy (real time x-ray imaging) have also been demonstrated though not yet commercialized [63]. The fluoroscopic application involves binning pixels to increase the signal-to-noise ratio.

The present review examines the ideal photoconductor requirements for a flat panel x-ray image sensor in Section 2 and then provides a critical comparison between various potential large area x-ray photoconductors in terms of their suitability. Section 3 examines how charge trapping in the photoconductor affects the sensitivity, the DQE and the resolution *i.e*., the MTF. We use a-Se as an example and compare its properties with other potential photoconductors. Section 4 outlines the dark current problem in large area x-ray photoconductors and how it was solved in the case of stabilized a-Se by the introduction of blocking layers at the electrodes. The reduction and control of the dark current was a milestone achievement in the development of a-Se based FPXIs [64]. In this section we also compare the dark current in different amorphous and polycrystalline layers. Section 5 discusses impact ionization in a-Se at sufficiently high fields, and how current research is likely to develop all solid state a-Se imaging devices that can exhibit large avalanche gains. Such photoconductors with avalanche gain have potential for use in medical imaging and would improve the DQE at low exposure. Throughout the paper, we emphasize how the overall sensor performance is closely related to the photoconductor material.

The present review only considers potential amorphous or polycrystalline photoconductors that can be or could be deposited on a large TFT-AMA substrate to fabricate a FPXI that would be useful in x-ray imaging such as radiography or fluoroscopy. Requirements for smaller area pixellated radiation detectors of the type used in radiation spectroscopy (where one is interested in measuring the incident photon energy) are quite different, and the reader is referred to other reviews in this area (e.g., [65,66]). Photoconductor coated imaging chips, such as photoconductor-coated CMOS imagers, offer additional functionality and speed compared with TFT-AMA based imagers. The main drawbacks of photoconductor-coated imaging chips are their limited area, that is, field of view requiring *tiling* for large area applications, and their cost.

## 2. Potential Large Area Photoconductors

It is important to clearly identify what are the photoconductor requirements for FPXI applications so that various candidate photoconductors can be compared and contrasted. Each clinical application will have different requirements, which implies that there is no single optimum. When an x-ray photon is absorbed in the photoconductor medium, as a result of the photoelectric effect, an energetic primary electron is knocked out from an inner core shell, for example the K-shell. The primary electron has a large kinetic energy given by *E*_ph_ − *E*_binding_, where *E*_ph_ is the x-ray photon energy and *E*_binding_ is the binding energy of the electron in the shell from which it was knocked out. As the energetic primary electron travels in the medium, it interacts with it and transfers energy to it, which results in the generation of many electron hole pairs, as well as phonons. The electron and hole pairs that are generated are those carriers that must be collected; phonons essentially represent losses. The applied electric field drifts the carriers to their respective electrodes for charge collection. The photoconductor effectively converts the incident radiation energy to electric charges, which constitute the signal.

Table 1 summarizes some of the properties of potential large area x-ray photoconductors that have shown promise for use in FPXIs. The table is a representative selection based on the fact that at least a prototype imager has been demonstrated and there is potential to scale up the imager for medical applications. Some of these have been recently discussed in the literature by other authors [16,67]. We first note that they are either amorphous or polycrystalline and often prepared by vacuum deposition techniques such as physical vapor deposition (PVD). In the following subsections we discuss the most important attributes of a photoconductor for FPXI applications.

### 2.1. Quantum Efficiency A_Q_

**Figure 5.**
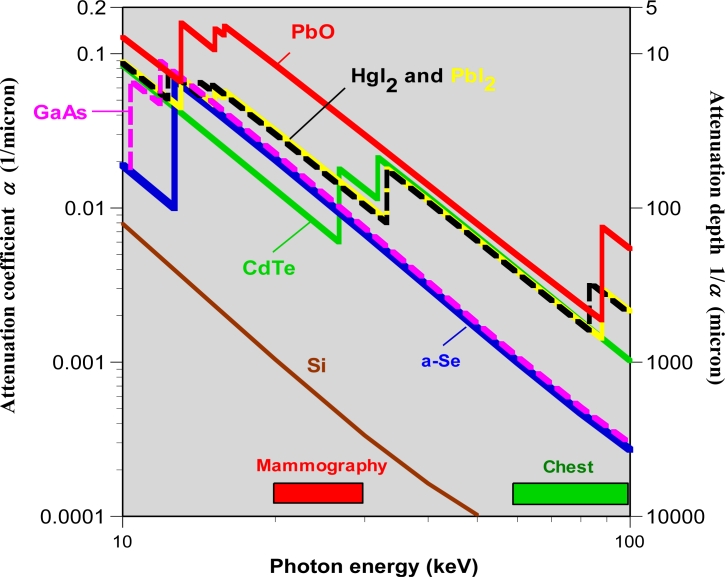
Linear attenuation coefficient *α* and depth, 1/*α* *vs.* photon energy for various photoconductors of interest.

Nearly all the incident x-ray radiation should be absorbed within a practical photoconductor thickness to avoid unnecessary patient exposure. Over the energy range of interest, the linear attenuation coefficient *α* must be sufficiently large to allow the incident photons to be attenuated inside the photoconductor. Put differently, the x-ray attenuation depth *δ*, the reciprocal of *α*, must be substantially less than the photoconductor layer thickness *L*. The fraction of incident photons in the beam that are attenuated by the photoconductor depends on *α* of the photoconductor material and its thickness *L*; and is given by

(1) $$A_{Q}=\text{Attenuated}\;\text{fraction}=[1-\text{exp}(-\alpha L)]$$

where *α* = *α* (*E*_ph_*,Z,* *ρ*) is a function of photon energy *E*_ph_, atomic number *Z* and density *ρ* of the material. *A_Q_* is called the *quantum efficiency* (QE) because it describes the efficiency with which the medium attenuates photons. The attenuation depth *δ* is where the beam has been attenuated by 63%. The law of diminishing returns arising from exponential absorption in Equation (1) indicates that a doubling of *L* can only result in a further 63% attenuation of the remaining beam (1 − 0.63 = 37%) for a total attenuation of 63 + 23 = 86%. Figure 5 shows the *α* *vs.* photon energy behavior for a number of x-ray photoconductors. The commercial mammographic detector in Figure 3 has an a-Se layer that is nominally 200 μm thick. At 20 keV (within the mammographic range), the attenuation depth is 49 μm so that the photoconductor’s *A_Q_* is 98.3%. At higher energies, for example at 60 keV, *δ* is 998 μm so that for an a-Se layer of thickness 1,000 μm, *A_Q_* is only 63.2%. Although increasing the thickness would increase *A_Q_*, it becomes practically very difficult to design sensors using thicker a-Se layers. It can be seen from Figure 5 that photoconductors with higher-Z components such as PbO, PbI_2_, HgI_2_, CdZnTe have very good quantum efficiencies in the high energy range that covers such modalities as chest radiography and angiography.

### 2.2. Electron-Hole Pair Creation Energy W_±_

We need the photoconductor to have as high intrinsic x-ray sensitivity as possible, *i.e.*, it must be able to generate as many collectable (free) electron hole pairs (EHPs) as possible per unit of absorbed radiation. The amount of radiation energy required, denoted as *W*_±_, to create a single *free* electron and hole pair is called the *electron-hole pair creation energy* or the *ionization energy*; and it should be as low as possible because the free (or collectable) charge, Δ*Q*, generated from an incident and absorbed radiation of energy Δ*E* is simply *e*Δ*E/W*_±_, where *e* is the elementary charge. For many material systems *W*_±_ is proportional to the bandgap *E_g_*. Indeed, for many crystalline semiconductors it is well-known that *W*_±_ ≈ 3*E_g_* (the so-called *Klein rule* [72,73]), which means that a lower *W*_±_ requirement suggests a semiconductor with a narrower bandgap. Unfortunately, narrow bandgap semiconductors do not have sufficiently low thermal equilibrium concentrations of carriers to result in a low dark current. a-Se, as in the case of various other low-mobility solids, is an exception to the Klein rule inasmuch as *W*_±_ decreases sharply with the applied electric field *F* and exhibits a behavior that follows [74,75]

(2) $$W_{\pm }=W_{\pm }^{\circ }+B/F$$

where $W_{\pm }^{\circ }$ is the intrinsic EHP creation energy at “infinite field”, *B* is a constant that weakly depends on the x-ray photon energy, and *F* is the applied field. In the 20–40 keV range one can simply take $W_{\pm }^{\circ }\approx 6\;\text{eV}$ and *B* ≈ 4.4 × 10^2^ eV·V μm^−1^, which makes *W*_±_ about 50 eV at *F* = 10 V μm^−1^. We should mention that a-Se's *W*_±_ at typical operating fields (10–20 V/μm) is poorer than competing large area x-ray photoconductors, as apparent from Table 1, such as HgI_2_ which has a *W*_±_ of 5 eV. However, while lower *W*_±_ is important in generating as much charge as possible from absorbed radiation, this charge still needs be collected. Although nearly all large area polycrystalline photoconductors have a lower *W*_±_ than a-Se (Table 1) they do not currently possess sufficiently good charge collection efficiency for both electrons and holes at an operating field that results in an acceptably low dark current. Consequently, the sensitivity of these polycrystalline photoconductors as gauged by the actual charge collected per unit incident radiation, may not necessarily be better. We have extensively discussed the behavior of *W*_±_ in the case of a-Se previously [17,19,75] and therefore will not address the origin of the field dependence of *W*_±_ further. *W*_±_, of course, enters the calculation of the overall x-ray sensitivity and DQE of the photoconductor; see Section 3.1

### 2.3. Dark Current

The dark current of the photoconductor under a bias voltage should be negligibly small. The dark current in most photoconductive semiconductors is normally attributed to one of two factors—the rate of injection of carriers from the contacts into the photoconductor and the rate of thermal generation of carriers. A small dark current implies that the contacts to the photoconductor should be non-injecting, and the rate of thermal generation of carriers from various defects or states in the bandgap should be negligibly small (*i.e*., dark conductivity is practically zero). Small dark conductivity generally requires a wide bandgap semiconductor that conflicts with the condition of smaller ionization energy. There have been several attempts to estimate what would constitute a negligible dark current density, *J_d_* [17]; it is generally accepted that *J_d_* should preferably not exceed 10 pA mm^−2^ depending on the clinical application. Section 4 examines in more detail what determines the upper limit on *I_d_*, how this was reduced in practice below the latter limit for a-Se, and our current understanding on the origin of the dark current.

### 2.4. Charge Collection Efficiency CCE

Once the charges are generated by the absorption of x-rays, these charges have to be collected. The applied field *F* shown in Figure 2 drifts the electrons and holes in opposite directions towards their respective electrodes. During charge transport we should not lose carriers due to recombination or trapping. Suppose that *τ* is the mean lifetime of a charge carrier, which could be due to recombination or deep trapping, and the drift mobility of the carrier is *μ*. Then *μτF* represents the mean distance drifted by the carrier before it is trapped (or disappears by recombination); this distance is called the *schubweg*. Since we need to collect most of the charges, both electrons and holes, we need to ensure that the electron and hole schubwegs are both much longer than the thickness of the photoconductive layer, that is, *μτF* ≫ *L* for both electrons and holes. If the photoconductor layer is made thicker to capture more of the radiation (towards increasing *A_Q_*), the *μτF* ≫ *L* condition would eventually be lost, and charge collection efficiency would start limiting the sensitivity. We discuss the charge collection efficiency and how it depends on the photoconductor properties in Section 3. The drift mobility × lifetime product, *μτ*, is normally called the *range* of the carrier, that is, its schubweg per unit field. Table 1 compares the carrier ranges among various large area x-ray photoconductors. One of the distinct advantages of a-Se is the fact that both electrons and holes possess reasonable ranges, which allows both electrons and holes to be collected upon their photogeneration. It is well known that a-Se exhibits typical “polymeric glass” properties in the sense that its properties relax (age) over time, see, for example, [76]. The carrier drift mobilites and lifetimes, and hence the carrier ranges, “relax”, that is, improve over time towards their equilibrium values following a typical stretched exponential behavior. For example, the deep hole and electron trap concentrations decrease as a-Se ages (relaxes), which results in the improvement of hole and electron lifetimes. The relaxation time scale is typically a few days, depending on the alloy composition [77].

In the case of polycrystalline semiconductors, the carrier ranges are highly dependent on the quality of the layer such as the grain size (polycrystallinity) and purity. For example, Schieber and Zuck quote maximum *μτ* values of 10^−3^ cm^2^/V for electrons and 10^−5^ cm^2^/V for holes in their best quality physical vapor deposited polycrystalline HgI_2_ layers [78]. These values would render HgI_2_ as one of the best polycrystalline x-ray photoconductors in terms of charge transport properties. The charge collection efficiency (CCE, *η*_CC_) is discussed further in Section 3 with its effects on the imager performance.

### 2.5. X-Ray Damage and Fatigue

One would expect that as the photoconductor is exposed to x-rays over time, its properties will deteriorate to some extent. Indeed, radiation damage is a well-known issue in material science. During the irradiation, the photoconductor structure itself can become either temporarily or permanently “*damaged*” by the generation of various defects. One can expect x-ray induced new defects as well as an increase in the population of certain intrinsic defects. These defect populations would try to return to their thermal equilibrium concentrations but the rate of “defect relaxation” is generally thermally activated (e.g., diffusion controlled) and its time scale therefore highly dependent on the activation energy. In some cases, defects anneal out when the photoconductor is left unexposed for a few days and in some cases the defects take much longer to anneal and may appear permanent over the observation time.

Another issue of significant importance is that as the photoconductor is subjected to repeated irradiation, or to a large dose, there will be a build-up of trapped charge carriers in the bulk of the photoconductor [79]. In the case of a-Se photoconductors, electrons are more likely to become trapped since they have shorter schubwegs than holes [80]. Electron release times from deep traps are very long, on the order of hours. Accumulation of trapped electrons results in two undesirable effects. First, newly photogenerated holes can recombine with previously trapped electrons and are thereby prevented from reaching the collection electrode. The latter results in a drop in the x-ray sensitivity of the exposed region of the x-ray sensor, and can lead to an effect called *ghosting* in x-ray imaging [81]. Secondly, the net space charge due to trapped carriers (e.g., electrons) modifies the internal field and hence modifies the charge collection efficiency. In the case of a-Se, the modified field leads to changes in the photogenerated number of carriers since *W*_±_ depends on the field. The modification of the internal field normally leads to a drop in the x-ray sensitivity [82]. In some x-ray photoconductors one species of carriers has a very limited range and becomes deeply trapped very quickly. The trapped carriers effectively polarize the sample, and modify the field and can reduce the charge collection efficiency [71,83].

### 2.6. Large Area Fabrication

One of the most important requirements for a large area x-ray photoconductor is that it must be capable of being coated to the required thickness over a large area to cover the AMA, e.g., 24 cm × 30 cm for mammography. The large area coating requirement obviously rules out the use of x-ray sensitive crystalline semiconductors which are difficult to grow in such large areas, and would require process temperatures incompatible with the glass AMA substrate and its a-Si:H electronics. Various polycrystalline semiconductors, such as Cd*_x_*Zn_1–x_Te (CZT), HgI_2_, PbI_2_, PbO, *etc.* as summarized in Table 1, have the feasibility to be prepared in large areas but their main drawback is the adverse affect of grain boundaries in limiting charge transport and, further, the high substrate and annealing temperatures required to optimize the semiconductor properties. High substrate temperature (in excess of 200 °C) are not compatible with a-Si:H TFT-AMA substrates onto which these polycrystalline semiconductors have to be coated. Nonetheless, much progress has been made in the last decade in preparing large area polycrystalline photoconductors that show promise in FPXI applications. For example, screen printed polycrystalline HgI_2_ does not need high substrate temperatures and has exhibited reasonable properties in terms of x-ray sensitivity [68].

Organic photoconductors currently dominate the xerographic photoreceptor industry (where they replaced a-Se and a-As_2_Se_3_) and can be cheaply prepared in large areas. However, they are of limited value for x-ray imaging because they do not absorb x-rays sufficiently well due to their poor attenuation coefficient arising from the very low *Z* elements comprising the organics (H, C, N, O). On the other hand, amorphous semiconductors such as a-Se, a-As_2_Se_3_ and a-Si:H are routinely prepared in large areas for such applications as xerographic photoreceptors and solar cells and are therefore well suited for flat panel x-ray detector applications. Amongst the three, a-Se is particularly well suited because it has a much greater x-ray absorption coefficient than a-Si:H, due to its greater atomic number, and it possesses good charge transport properties for both holes and electrons compared with a-As_2_Se_3_ in which electrons become trapped and the hole mobility is much smaller. Further, the dark current in a-Se is much smaller than that in a-As_2_Se_3_. Table 1 provides a summary of typical preparation procedures for some selected x-ray photoconductor materials. Due to its commercial use as an electrophotographic photoreceptor, a-Se is one of the most highly developed photoconductors [15]. It can be easily coated as thick films (e.g., 100–500 μm) onto suitable substrates by conventional vacuum deposition techniques and without the need to raise the substrate temperature beyond 60–70 °C. Its amorphous state maintains uniform characteristics to very fine scales over large areas.

### 2.7. Speed

There is a need to use the FPXIs in real time applications such as fluoroscopy. Once the electrons and holes are photogenerated by the absorption of x-rays, they should then drift within a reasonable time and become collected. The collection time for the x-ray generated charge is limited by the slowest carriers, that is, the carrier that has the lowest drift mobility. In the case of a-Se (assuming stabilized a-Se), electrons have a drift mobility that is roughly 2 × 10^−3^ cm^2^ V^−1^ s^−1^ and much lower than that for holes. Taking a worst case calculation, the electron transit time across a 1,000 μm layer (assuming, for example, a fluoroscopic application in which the a-Se layer has to be reasonably thick to absorb the x-rays) is typically 0.5 ms under an applied field of 10 V μm^−1^. This is much shorter than the shortest interframe time (33 ms for 30 fps) that one can expect to encounter in various common x-ray imaging applications. In the case of the mammographic sensor described above, with a thickness of 200 μm, the electron transit time is shorter than 0.1 ms.

## 3. Charge Carrier Transport and Imager Performance

Once the absorbed x-rays are converted into electrons and holes, these carriers have to be drifted and collected. One of the most important issues in current large area photoconductor problems is the limitation imposed by insufficiently long carrier schubwegs, that is, not all the photogenerated charges are collected. Table 1 lists some of the carrier ranges that have been reported for those x-ray photoconductors we have been thus far considering. For some of the polycrystalline photoconductors there is a very large disparity between the ranges of the two types of carriers; that is, while one type of carrier has a long range and becomes collected, the other type is likely to be trapped and not be collected. For example, in polycrystalline PbI_2_, electrons have poor ranges and become trapped while holes can be easily collected.

**Figure 6.**
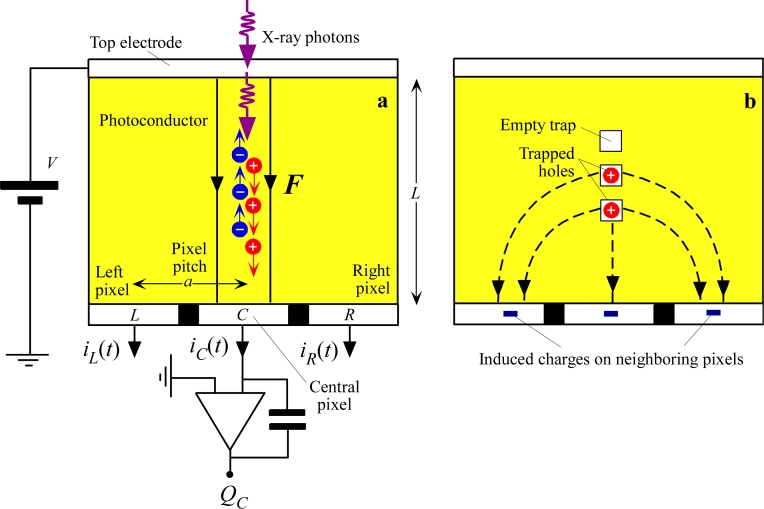
**(a)** X-ray photons are incident on a central reference pixel *C* and are absorbed in the photoconductor over *C*. The x-ray generated electrons and holes drift respectively towards positive and negative electrodes, the latter being pixellated. There is no trapping and recombination and all the generated charges are collected. **(b)** Holes are trapped. These trapped charges result in a loss of sensitivity and resolution.

Consider a photoconductor biased positively on the radiation receiving electrode as illustrated in Figure 6(a). Suppose that x-ray photons are incident along a line through the centre of some reference pixel, labeled as *C* for “central”. These photons become absorbed in the photoconductor over *C* and generate electrons and holes that drift towards positive and negative electrodes respectively. While the carriers are drifting there are induced currents at the pixels of the bottom electrode, for example on the left *L* and right *R* of the reference pixel *C.* (Remember that in reality this is a two dimensional array). If we integrate the transient current that flows out from a particular pixel, for example, pixel *C*, we would find the charge collected *Q_C_* at that pixel, which is the pixel of interest, *C*. The charges collected at the three neighboring pixels *L*, *C* and *R* in Figure 6(a) are

(3) $$Q_{L}=\int i_{L}(t)dt;\;\;Q_{C}=\int i_{C}(t)dt;\;\;Q_{R}=\int i_{R}(t)dt$$

where the integration is longer than the exposure time plus the longest transit time. In the absence of any trapping and loss of carriers, *Q_C_* would be equal to the charge generated by the absorbed x-ray radiation, that is *Q_C_* = *eE*_absorbed_/*W*_±_, where *E*_absorbed_ is the amount of absorbed radiation energy. The charges *Q_L_* and *Q_R_* would be zero even though there were transient currents *i_L_*(*t*) and *i_R_*(*t*) flowing at these pixels during the drift of the carriers. Indeed, there is a change in the sign of the current flows at *L* and *R* so that when *i_L_*(*t*) and *i_R_*(*t*) are integrated they result in *Q_L_* = *Q_R_* = 0 [84].

However, if holes are lost during their drift by capture into deep traps from which there is no escape over the time scale of interest, as shown in Figure 6(b), then there are two immediate effects. First, less charge is collected and hence there is a *reduction* in the sensitivity [85–88]. This effect is termed *charge collection efficiency* limited sensitivity. Secondly, the trapped holes will induce negative charges on neighboring pixels, and hence spread or *blur* the information. Put differently, the charges *Q_L_* and *Q_R_* will not be zero but finite. The latter effect is a loss of resolution. It should be emphasized that the drifting holes actually induce transient currents not only on their own pixel (*C*) but also on neighboring pixels, *L* and *R* in Figure 6 [89]. Table 2 summarizes some of the recent treatises that have addressed the effects of trapping on the sensitivity, DQE and MTF. The reader should note that the majority of the work relating the x-ray image detector performance to charge carrier deep trapping effects was done in Canada. For example, the papers by Mainprize, Hunt and Yaffe [90] in 2002 and Kabir and Kasap [87,91] 2002–2003 examine the effects of incomplete charge collection on the detector performance. In a Medical Physics paper published in 2005, Rowlands and coworkers were able to attribute the ghosting in a-Se detectors primarily to the recombination of holes with previously trapped electrons [81] when the latter is significant. Most recently, Kabir, Kasap and Rowlands's groups jointly explained the changes in the MTF, the resolution, of a-Se detectors upon repeated exposure in terms of charge carrier trapping effects [92]. Currently, the work that relates detector performance to charge trapping effects continues as an important topical research area within three main photoconductor groups within Canada; Rowlands and Reznik at Lakehead University, Kabir at Concordia University, and Kasap at the University of Saskatchewan.

It is useful to mention that the effects of charge trapping in pixellated *crystalline* detectors, especially those used in spectroscopic measurements, have been studied in detail by a number of researchers; two recent examples are [93,94]. In the present case, we are interested in the effects of charge trapping in amorphous (a-Se) and polycrystalline photoconductors and how trapping affects the imaging performance *viz* sensitivity, DQE and MTF. While some of the effects are similar, there are differences in the way in which photoconductor material can be modeled, and the performance metric of interest, e.g., DQE and MTF *vs.* energy resolution.

### 3.1. X-Ray Sensitivity

The overall conversion efficiency of incident radiation to collected charge relies on three distinct processes. First is the attenuation of the x-rays in the photoconductor, determined by *A*_Q_, and the absorption of the radiation energy, determined by (*α*_en_/*α*)*E*_ph_, per attenuated photon in which *α*_en_ is the energy absorption coefficient at the energy of interest. The second process is the conversion of absorbed radiation to electron and hole pairs, which is determined by ionization energy *W*_±_; the so-called electron and hole creation energy. The third process is the drift and eventual collection of the photogenerated charge carriers, the efficiency of which is determined by *η*_CC_. It is useful to define the x-ray sensitivity, *S*_x_, of a photoconductor as the charge collected per unit incident radiation per unit area. The incident radiation is the x-ray exposure measured in Roentgens; *S*_x_ would be C m^−2^ R^−1^ in SI units. At one specific photon energy *E*_ph_,

(4) $$S_{x}=\left(\frac{5.45\times 10^{13}e}{{\left(\alpha_{\text{en}}/\rho \right)}_{\text{air}}}\right)\times A_{\text{Q}}\times \left(\frac{(\alpha_{\text{en}}/\alpha )E_{\text{ph}}}{W_{\pm }}\right)\times \eta_{\propto }$$

where the first term gives the incident photon fluence per unit Roentgen, the second is the attenuated fraction of the incident photons, that is, the quantum efficiency, the third is the number of EHPs created per absorbed radiation energy, and the fourth is the fraction of those charges that are actually collected. The quantity (*α*_en_/*ρ*)_air_ is the energy absorption coefficient per unit density for air. All terms in Equation (4) depend on the photon energy and, for most semiconductors, only the last term, *η*_CC_, depends on the applied field. In the case of a-Se, the third term that represents the x-ray intrinsic photogeneration yield also depends on the electric field via *W*_±_.

It is important to emphasize that a meaningful comparison between competing photoconductors must include using the appropriate values for all four factors in Equation (4). For example, one cannot simply assume full charge collection efficiency and simply compare *A_Q_* and *W*_±_ among various photoconductors. For many photoconductors one has to accept a compromise between the maximum dark current that can be tolerated and the maximum field that can be applied, which significantly reduces *η*_CC_ and hence reduces the overall sensitivity of the photoconductor of interest.

It is instructive to examine the expression for *η*_CC_ first for a photoconductor in which the bottom electrode is not pixilated, the simplest case [85,86]. For the radiation receiving electrode biased negatively [85–87]

(5) $$\begin{array}{c} \eta_{\text{CC}}=\eta_{\text{HCC}}+\eta_{\text{ECC}}=x_{h}\left(1-\frac{1-\text{exp}(-1/\Delta -1/x_{h})}{\left[1+(\Delta /x_{h})][1-\text{exp}(-1/\Delta )\right]}\right)\\ +x_{e}\left(1-\frac{\text{exp}(-1/x_{e})-\text{exp}(-1/\Delta )}{\left[1-(\Delta /x_{e})][1-\text{exp}(-1/\Delta )\right]}\right) \end{array}$$

where Δ = *δ/L* is the normalized attenuation length, *x_h_* *=* *μ_h_τ_h_F*/*L* is the schubweg per unit sample thickness for holes, *x_e_* = *μ_e_τ_e_F*/*L* is that for electrons, and *η*_HCC_ and *η*_ECC_ are the CCE for holes and electrons respectively. Clearly, the CCE depends on the photoconductor thickness *L* and decreases as the thickness increases while keeping the field the same. On the other hand, *A_Q_* increases with increasing thickness. There is therefore an optimum thickness beyond which the sensitivity decreases [86]; the latter depends on *α* at the photon energy of interest, the charge carrier ranges (*μτ*) and the operating field *F*. One further consideration is that maintaining the same field in thicker photoconductors would require greater voltages to be applied and there may be practical limits to the applied bias voltage. For example, if we choose an a-Se photoconductor with a thickness of 1,000 μm to absorb 95% of the photons of energy around 40 keV (where *δ* = 325 μm, *L*/3), the required applied voltage is 10 kV to maintain the operating field at 10 V μm^−1^. There would obviously be practical challenges in applying higher voltages within the detector electronics.

**Figure 7.**
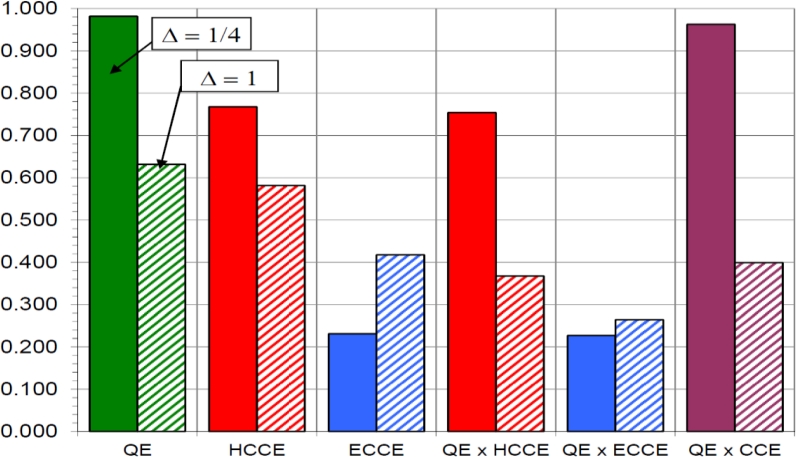
The comparison of *A_Q_* (QE) and *η*_CC_ (CCE) contributions to the x-ray sensitivity for two different attenuations, *i.e*., for two different photon energies. Full colors are for Δ = 1/4 (*δ* = *L*/4) and hatched colors are for Δ = 1 (*δ* = *L*). HCCE and ECCE are the hole and electron collection efficiencies respectively. The radiation receiving electrode is positively biased. For a unipolar semiconductor either HCCE or ECCE would be zero.

Figure 7 compares the contributions of the quantum *A_Q_* and *η*_CC_ contributions to the x-ray sensitivity for two different attenuations, *i.e*., for two different photon energies. HCCE and ECCE are the hole and electron collection efficiencies respectively. The case for Δ = 1/4 represents a photoconductor that attenuates the radiation reasonably well (Δ < 1). The most important carriers to collect are those moving towards the bottom electrode, which contribute 76.8% to the CCE whereas those traveling towards the top electrode make a contribution of only 23.2%. When Δ = 1, on the other hand, these contributions become 58.2 and 41.8% respectively; in such a case, the photoconductor selection and design must aim to collect both types of carriers to avoid an excessive loss of sensitivity.

While the sensitivity discussion above is useful, it is further complicated by having the bottom electrode pixillated so that small pixel effects become important [84]. Suppose that we are interested in the x-ray sensitivity of the pixel *C* in Figure 6(a) to the absorbed radiation just above this pixel, that is, if the charge collected at *C* is Δ*Q_C_* and the x-ray radiation absorbed above *C* is Δ*X* and the area of pixel *C* is *A*, then *S_x_*(*C*) = Δ*Q_C_*/*A*Δ*X*. During the drift of the carriers, charges will be induced at both neighboring pixels and we need to know the weighting potential distribution to properly calculate the collected charge at *C*. The procedure relies on the Shockley-Ramo theorem to find the induced charges at the pixels due to the drift of carriers above the pixels. The following important conclusions come out from the analysis, assuming that the radiation receiving electrode is positively biased as in Figure 6(a). As the pixel size shrinks with respect to the photoconductor thickness, the sensitivity *S_x_*(*C*) becomes much more sensitive to the trapping of holes and *S_x_*(*C*) is actually lower than one would expect for an unpixellated sensor. On the other hand, as the pixel size shrinks, the sensitivity is less affected by electrons, which drift towards the top electrode. Thus, it is essential to ensure that carriers that are drifting towards the pixel *C* have good transport properties. Put differently, the sensitivity can be improved by ensuring that the carrier with the higher mobility-lifetime product is drifted towards the pixel electrodes; a general treatment with an application to a-Se may be found in [96,97].

When a photoconductor is repeatedly exposed to radiation or is subjected to a large dose, its sensitivity tends to decrease; a phenomenon known as *x-ray fatigue*. Further, there can be localized changes in the sensitivity due to a previous exposure. Such localized changes can give rise to image *ghosting* as mentioned above. The reduction in the sensitivity *S_x_*, according to Equation (5), can be due to a fall in *η*_CC_ (CCE) and/or a decrease in the photogeneration efficiency (1/*W*_±_). The fall in the CCE can be due to the creation of more traps by the absorbed radiation, the recombination of drifting carriers with previously trapped oppositely charged carriers or other factors. Trapped carriers create a bulk space charge which modifies the field and changes the effective *W*_±_, if the latter depends on the field as in a-Se. A recent study clearly high-lights these effects on the sensitivity in the case of a-Se which has been exposed to a large dose [98].

### 3.2. Detective Quantum Efficiency

A meaningful comparison of different photoconductive sensors must involve the evaluation of DQE, as a function of spatial frequency, *f*, which is a task that is not trivial inasmuch as we must be able to identify and quantify all noise contributions in the imaging chain from input to the output. DQE is defined as

(6) $$\text{DQE}(f)=\frac{{\text{SNR}}_{\text{out}}^{2}(f)}{{\text{SNR}}_{\text{in}}^{2}(f)}$$

where SNR_in_ and SNR_out_ are the signal to noise ratio at the input and output stages of the detector, respectively. DQE(*f*) is considered as an appropriate metric of system performance to compare with model calculations; and is unity for an ideal detector. This is only true however for a linear system or one that can be linearized. One example of a non-linearlizable system is one with aliasing, so care needs to be used if aliasing occurs which is quite likely, perhaps inevitable for a photoconductor based system. We are often interested in the zero spatial frequency detective quantum efficiency DQE (*f* = 0) of an imaging detector. DQE(0) represents signal quality degradation due to the signal and noise transfer characteristics of the system without considering signal spreading. The signal and noise transfer through an x-ray image detector is a complex process.

A cascaded linear-system model has been used by various investigators to characterize the performance of many imaging systems in terms of signal transfer and noise-transfer relationships from input to the output through various stages, taking into account significant noise sources, [30,99,100], with applications to a-Se [90,91] and PbO [101] detectors.

**Figure 8.**
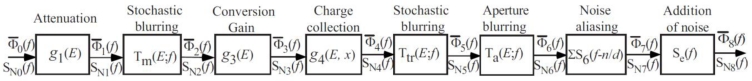
An example of one simple linear cascaded systems model recently used in modeling the DQE of a PbO FPXI, which neglects K-fluorescence reabsorption. After [101].

In the cascaded linear systems model, an imaging system is described as cascades of simple and independent elementary stages. The input and the output of each stage are distributions of quanta. The random nature of image-related quanta creates statistical fluctuation in image signals contributing to image formation. The noise in the number of x-rays, or signal incident on the detector, is given by a *Poisson fluctuation*. For example, if the mean incident x-ray fluence on a detector is Φ̅_0_ photons per unit area, the input noise power spectrum (NPS) in the number of x-rays incident on the detector is given by, *S_N_*_(0)_ = Φ̅_0_ as determined by Poissons statistics. The signal and noise are passed through various stages in an imaging system which can be classified into five processes: (a) gain, (b) stochastic blurring (c) deterministic blurring, (d) aliasing and (e) the addition of noise. For example, for PbO FPXIs [101] the K-fluorescence can be neglected and one can use a cascaded linear system model that has eight states *i.e.*, x-ray attenuation, scattering of x-ray photons before the photoelectric effect, photogeneration of charge carriers (conversion gain), charge collection, blurring due to charge trapping, aperture blurring, noise aliasing and the addition of electronic noise as shown in Figure 8.

In the linear cascaded systems model, at each stage *i* the signal Φ̅*_i_* and NPS S*_N_*_(_*_i_*_)_ are calculated from the signal and NPS of the previous stage. For example, for a gain stage, fluctuations in its transfer characteristics results in a mean gain *g̅_i_* and a variance $\sigma_{g_{i}}^{2}$. The output mean signal quanta per unit area (Φ̅*_i_*) and NPS (*S_N_*_(_*_i_*_)_ (*f*)) are given by [100]

(7) $${\bar{\Phi }}_{i}={\bar{g}}_{i}{\bar{\Phi }}_{i-1}$$

(8) $$S_{i}(f)={\bar{g}}_{i}^{2}S_{i-1}(f)+\sigma_{g_{i}}^{2}{\bar{\Phi }}_{i-1}$$

where *f* is the spatial frequency, Φ̅*_i_*_–1_ and *S_N_*_(_*_i_*_–1)_ (*f*) are the mean number of quanta and the NPS incident on stage *i*, respectively, and *g̅_i_* and *σ_gi_*^2^ are the mean gain and variance of the gain of the *i*th stage. According to Equation (8), it is obvious that the stochastic amplification increases the noise associated with the quanta in two ways. First, the quantum noise is itself amplified, and secondly, noise associated with the stochastic nature of the amplifying mechanism is introduced into the output. Stochastic and deterministic blurring stages have their own representative equations that relate the output, Φ̅*_i_* (*f*) and *S_N_*_(_*_i_*_)_ (*f*), to the input, Φ̅*_i_*_–1_ (*f*) and *S_N_*_(_*_i_*_–1)_ (*f*) [99,100]. The aliasing stage represents the increase in the noise from the aliasing effect *i.e.*, the signal at spatial frequencies above the Nysquist frequency *f*_N_ can generate noise below *f*_N_ by aliasing [28]. In the noise addition stage, the noise from the electronics is added to the input noise. Eventually Φ̅_output_ and *S_N_*_output_ are calculated and hence the DQE as a function of spatial frequency. A more rigorous modeling of an imaging system usually also involves various parallel chains corresponding to other processes such as K-fluorescence reabsorption [97].

**Figure 9.**
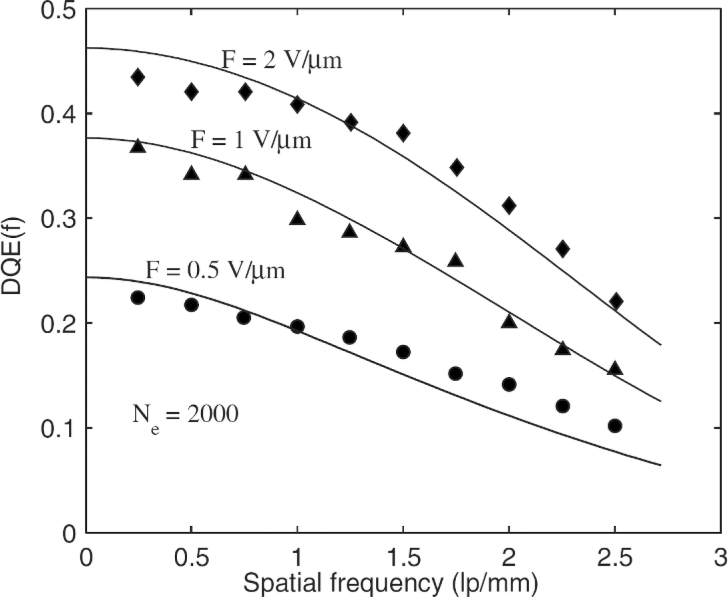
DQE(*f*) *vs.* spatial frequency *f* for a negatively biased PbO photoconductive x-ray sensor at three different applied fields, *F* = 0.5 V μm^−1^, 1 V μm^−1^, and 2 V μm^−1^. The symbols are the experimental points reported by Simon and coworkers and the solid line is the calculations based on a cascaded linear system model; further details may be found in [60,101].

The PbO case was examined recently by Kabir at Concordia University in Montreal and it is worth mentioning [101]. There is a cascade of stages that represent the x-ray attenuation, conversion, charge collection, blurring due to incomplete charge collection, and the addition of electronic noise. The work considers both stochastic and deterministic blurring and includes the blurring effect of the photoelectron range. The K-fluorescence reabsorption is not too significant, which makes the DQE modeling easier. The final result of the calculations and the comparison with the experimental DQE *vs.* spatial frequency *f* data is shown in Figure 9. The most important factor that affects the DQE in the case of PbO was attributed to the charge trapping effects, that is, insufficient charge collection efficiency. Kabir neglected any conductance fluctuations of the photoconductor, that is, noise arising from fluctuations in the thermal generation rate of carriers in the bulk of the semiconductors in Figure 8. We believe that the latter would not change the conclusions of the work in Figure 9. Similar DQE analysis of a-Se shows that trapping or insufficient charge collection can also lead to the lowering of the DQE [91,97] and necessitates a good quality control on the charge transport properties of the photoconductor material from which the detector is fabricated.

### 3.3. Resolution and Modulation Transfer Function (MTF)

As mentioned above and as is apparent in Figure 6(b), charge trapping effects can lead to charges being induced on neighboring pixels and hence affect the resolution of the sensor. The resolution is normally measured in terms of the modulation transfer function, MTF, which is the efficiency of an imaging system to resolve (transfer) different spatial frequencies of information in an image. In other words, MTF is the relative signal response of the system as a function of spatial frequency *f*. MTF can be defined at a given spatial frequency *f* by comparing the contrast at the output with that at the input when the input is an image pattern that has a sinusoidal spatial variation with a frequency *f*, *i.e*.,

(9) $$\text{MTF}(f)=\frac{\text{Image}\;\text{contrast}\;\text{at}\;\text{the}\;\text{output}\;\text{at}\;\text{spatial}\;\text{frequency}\;f}{\text{Image}\;\text{contrast}\;\text{at}\;\text{the}\;\text{input}\;\text{at}\;\text{spatial}\;\text{frequency}\;f}$$

MTF(*f*) and DQE are related by [102]

(10) $$\text{DQE}(f)={\bar{q}}_{0}G^{2}\frac{{\text{MTF}}^{2}(f)}{\text{NPS}(f)}$$

where NPS(*f*) is the noise power density spectrum of the output image, *q̄*_0_ is the average quanta incident onto the detector per unit area, and *G* is the detector gain.

**Figure 10.**
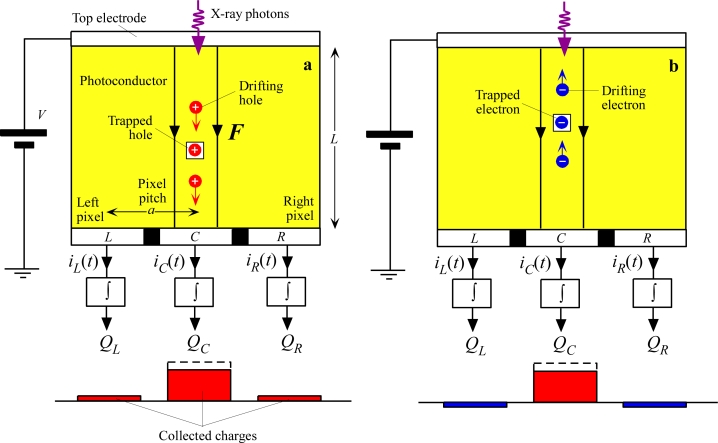
The effects of charge trapping on the resolution (MTF) depends on the type of carriers that have been trapped; whether carriers were drifting to the top or bottom electrode. *C* is the central (reference) pixel and *L* and *R* are the neighboring left and right pixels. The transient currents flowing into the pixels are integrated and eventually yield the collected charges at the pixels.

The actual effect on the MTF depends on the type of carrier that is trapped, *viz.* whether the carriers moving towards the top (radiation receiving) or bottom (pixellated) electrode are trapped. Charge trapping effects on the MTF of large area photoconductive detectors have been studied in detail by a number of researchers with applications to a-Se, CdZnTe and PbO based detectors [92,97,101,103]. The trapping of charges moving towards the bottom electrode, holes in Figure 10(a), results in the deterioration of the MTF at higher spatial frequencies, reducing the resolution. Intuitively, the drifting holes in Figure 10(a) induce currents in neighboring pixels, and result in pixels *L* and *R* registering charges*, Q_L_* and *Q_R_*, as apparent in Equation (3), which eventually become zero when the holes reach the central pixel *C*, and all the charge is collected on *C*. The trapping of holes results in charges *Q_L_* and *Q_R_* not diminishing to zero, as the holes are suddenly removed by capture into deep traps. Since *Q_L_* and *Q_R_* have the same sign as *Q_C_*, the information has been spread further away from *C* and results in the drop in MTF at higher spatial frequencies.

If electrons (carriers moving towards the top electrode) are trapped, then the situation on *L* and *R* are quite different since the induced charges on *L* and *R* now have an opposite sign to that on *C*. The integration of the induced currents flowing into *L* and *R* result in *Q_L_* and *Q_R_* having an opposite sign to the collected charge *Q_C_* on *C*, and hence the information appears “squeezed” towards *C*. There is an actual improvement in the high frequency response. In both cases, there is a reduction in the sensitivity with respect to that in which there is no trapping but the effects on the MTF are not the same. Obviously in the actual detector both carriers can become trapped and, further, there will also be other factors that make a contribution to the overall MTF. The exact theoretical treatment of the MTF in the presence of charge trapping may be found in [103] where normalized universal curves are given so that the model can be applied to any direct conversion FPXI. Conversely, one can adjust the *μτ* ranges of the carriers to match the model and experimental MTFs and then check whether these *μτ* values correspond to what are typical for the photoconductor material; in some cases independent measurements of *μτ* are available.

**Figure 11.**
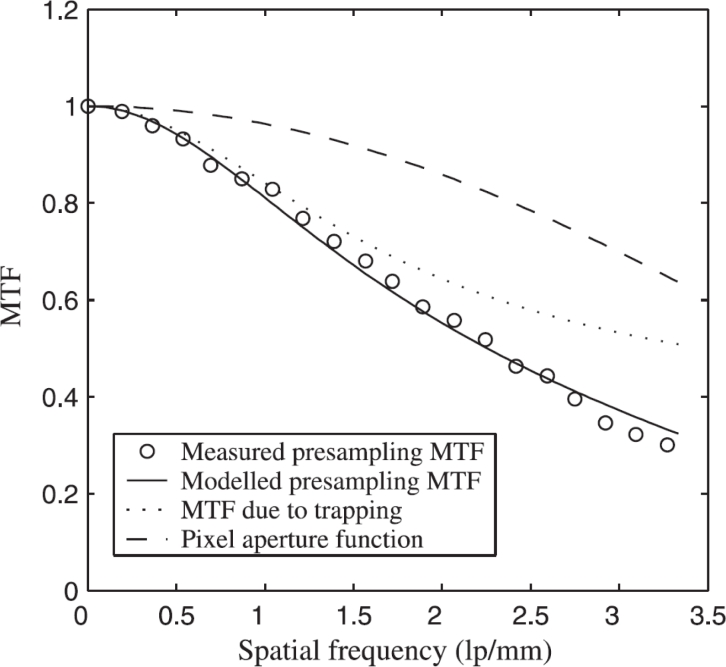
Measured presampling MTF *vs. f* of a polycrystalline CdZnTe detector in comparison with a calculated MTF in which deep trapping of charge carriers is included in the model. Blurring due to charge carrier trapping in the bulk of the photoconductor cannot be neglected. The detector thickness is 300 μm and the pixe pitch is 150 μm. After [103]. Data from [104].

Figure 11 shows a case study that involves a polycrystalline CdZnTe detector whose MTF has been measured [104]. The photoconductor thickness was 300 μm and the pixel size was 150 μm; the Nyquist frequency is 3.3 lp/mm. The operating electric field is 0.25 V/μm with the receiving electrode biased positively. The hole and electron ranges were adjusted until the model matched the MTF measurements. The best fit *μτ* products of electrons and holes are *μ_e_τ_e_* = 2.4 × 10^−4^ cm^2^/V and *μ_h_τ_h_* = 3.2 × 10^−6^ cm^2^/V, which are not too different than typical *μτ* values reported in the literature [105,106]. In fact, Mainprize *et al.* [105] reported a value of *μ_e_τ_e_* ≈ 2.4 × 10^−4^ cm^2^/V for polycrystalline CZT by modeling the charge collection efficiency (not MTF), which is in remarkable agreement with the value for *μ_e_τ_e_* from MTF modeling even though the two samples are different.

In the case of a-Se based mammographic FPXIs, the quality of the phoconductor is such that there is very little trapping in the photoconductor layer (*μτF* ≫ *L*). MTF measurements on a Siemens a-Se FPXI (Mammomat Novation DR with a pixel size of 70 μm) essentially produce an MTF *vs. f* behavior in which the dominant shape is a sinc function and close to the theoretical limit of sinc(*af*), where *a* is the pixel aperture; *i.e*., the MTF corresponds to what one expects from an ideal pixellated sensor with a pitch *a* [107].

Repeated use of a FPXI, or exposure to large doses can degrade the performance and can lead to a reduction in sensitivity, as explained above (Table 2). In addition there may be concurrent changes in the MTF. In one recent study, an a-Se FPXI with a thick (1 mm) photoconductor layer and biased negatively was examined for such effects [92]. A large exposure (1R) was first applied to cause the charge carrier trapping and also generate “new traps”. This is the ghosting dose. Following the ghosting dose, the sensitivity and the MTF were measured. The experiments were done at different fields as well so that the charge carrier schubwegs could be varied. The results clearly showed that there is a reduction in the sensitivity (which leads to ghosting) but there is an improvement in the high frequency region of the MTF. The results could be easily interpreted by applying the MTF model in [102]. This model involves charge carrier trapping, recombination of drifting carriers with previously trapped oppositely charged carriers, creation of new deep traps, the perturbation of the internal field by trapped charges, and the modification of charge transport and trapping as well photogeneration by the perturbed internal field. The charge carrier ranges (*μτ*) for holes and electrons in the MTF model was the same as those used in the sensitivity model, and corresponded to realistic values that could be verified independently by other measurements e.g., interrupted field time-of-flight (IFTOF) transient photoconductivity measurements [108].

The trapping of charge carriers considered above in the case of a-Se detectors can occur not only in the bulk of the photoconductor but also at the interface between the photoconductor and the blocking layer and/or within the blocking layer itself. The thickness of the blocking layer can therefore affect the MTF as shown by Hunter *et al.* [109].

In the above treatise we have primarily considered the effects of carrier trapping. It should be emphasized that there are several other factors that also influence the resolution besides the trapping of the charge carriers, depending on the photoconductor material [18,110]. In the absence of any deep trapping, and neglecting oblique incidence of x-rays, the main factors that would influence the MTF are expected to be the following. *K-fluorescence* involves the emission of a secondary K-fluorescence x-ray photon that is reabsorbed in a region away from the initial x-ray interaction point and hence causes blurring [111,112]. The creation of electrons and holes (ionization) occurs along the track of the primary electron, that is, the photoelectron that has been emitted. The primary electron track length is therefore also important and depends on the photon energy and the medium. If the track is long, it may generate carriers that can eventually overlap to the next pixel.

In the case of crystalline and some polycrystalline semiconductors, the charge carriers can diffuse laterally during their drift. The lateral diffusion of carriers would spread or blur the information. The effects of carrier diffusion in crystalline semiconductor based image detectors have been modeled, for example in [113,114]. We can easily estimate the effect of diffusion. Referring to Figure 6(a), the transit time *t_t_* of the carriers across the whole photoconductor thickness is *L*/*μF* or *L*^2^/*μV*^2^ where *μ* is the drift mobility. If *D i*s the diffusion coefficient of the carriers (*D/μ* = *kT*/*e*, where *k* and *T* are the Boltzmann constant and the temperature), then, in this time *t_t_*, the carriers diffuse laterally a root mean square (RMS) distance Δ*y*_diffusion_, given by $\Delta y_{\text{diffusion}}^{2}=\bar{\Delta y^{2}}=2Dt_{t}=2(\mu kT/e)(L^{2}/\mu V)=2L^{2}(V_{\text{th}}/V)$, where *V*_th_ = *kT/e* is the thermal voltage. For diffusion to be negligible, we need Δ*y*_diffusion_ ≪*a*/2, the half pixel size as we assumed the carriers are photogenerated along a line passing through the center of the pixel. The ratio Δ*y*_rms_/(*a*/2) is

(11) $$\frac{\Delta y_{\text{diffusion}}}{\left(a/2\right)}=2\sqrt{2}\left(\frac{L}{a}\right){\left(\frac{V_{th}}{V}\right)}^{1/2}$$

Thus the effect of diffusion on the sensor resolution can gauged by the relative magnitude of $V_{\text{th}}^{1/2}/a$ with respect to *V*^1/2^/*L*. Clearly, diffusion is important in the case of small pixel sensors (small *a* values) and also when the applied voltage is not sufficiently large. In the case of a-Se, *L/a* ≈ 2 and *V*_th_/*V* = (0.025 V)/(2 × 10^3^ V) so that Δ*y*_diffusion_/(*a*/2) ≈ 4 × 10^−2^, and the contribution of diffusion is negligible. This may not be the case with some of the other polycrystalline semiconductors which have to be operated at lower voltages to keep the dark current low.

The scattering of the x-rays, for example by Compton scattering, becomes more pronounced at higher energies, and can cause additional blurring. All these latter effects deteriorate the MTF of the sensor and have to be considered in detector design. In the particular case of a-Se, K-fluorescence and carrier trapping related effects seem to dominate the intrinsic MTF, that before the imposition of the aperture's sinc function.

Recently, Kim *et al.* considered the intrinsic DQE and the MTF of six x-ray photoconductors, a-Se, CdZnTe, HgI2, PbO and TlBr, for mammographic detector applications by neglecting charge trapping effects but including the thermal generation of carriers in the photoconductor, which is a noise source [10]. The Monte Carlo model shows clearly that all are highly suitable and exhibit an MTF greater than 0.75 up to spatial frequencies of 30 cycle mm^−1^, which probably represents the intrinsic (upper limit) resolution of these photoconductors. Once charge carrier trapping is introduced, however, distinct differences arise between the photoconductors [97].

## 4. Dark Current

As mentioned in Section 2.3, in an ideal detector, the dark current would be negligibly small. An unacceptably large dark current would cause a number of problems [17]. The noise, *i.e*., fluctuations in the large dark current, would create noisy pixels (poor signal-to-noise ratio). Such a dark current will constrain the dynamic range by allowing the voltage on the pixel capacitance to build up. Quite often, due to charge carrier trapping and polarization effects, the dark current decays with time from the instant of application of the bias voltage. It is a function of time and the applied nominal field. It also depends on the x-ray exposure. There are therefore variations in the dark current from pixel to pixel. Thus a large dark current, depending on prior exposure, is difficult to correct for and would lead to marked variations in the pixel SNR. Further, in rapid imaging, such as tomosynthesis, there is no time between readouts to correct for adverse effects of the dark current on the sensor performance; and hence the dark current limit is even more stringent.

The acceptable dark current depends on the exact application, though values in the range 1–10 pA mm^−2^ or 0.1–1 nA cm^−2^ are often quoted [17]. A rough intuitive argument can be made as follows. Suppose that *X*_ph_ is the minimum incident radiation signal (in R) that we wish to detect, which corresponds to Φ_ph_, the number of incident photons per unit area. The input quantum noise will be (Φ_ph_ *A*)^1/2^, where *A* is the pixel area. If the photon energy is *E*_ph_, then the number of charge carriers collected from the absorption of all (Φ_ph_ *A*)^1/2^ “noise” photons would be (Φ_ph_ *A*)^1/2^ (*α*_en_ / *α*) *E*_ph_ / *W_±_*, where *α*_en_ and *α* are the energy absorption and linear attenuation coefficients of the photoconductor. The dark current *I_d_* is integrated over a time, *t*, on the pixel capacitance so that it accumulates *N_d_* number of carriers, *N_d_* = *I_d_t*/*e*. The noise (fluctuations) in *N_d_* would be $N_{d}^{1/2}$ assuming the dark current evinces only shot noise. We would like the dark current density *J_d_* = *I_d_*/*A* to be such that $N_{d}^{1/2}\ll {\left(\Phi_{\text{ph}}A\right)}^{1/2}(\alpha_{\text{en}}/\alpha )E_{\text{ph}}/W_{\pm }$ or

(12) $$\sqrt{J_{d}At/e}\ll \frac{{\left(\Phi_{\text{ph}}A\right)}^{1/2}E_{\text{ph}}(\alpha_{\text{en}}/\alpha )}{W_{\pm }}$$

We can now substitute typical values for an a-Se sensor designed for mammography, and assuming the lower exposure *X*_ph_ is roughly 0.1 mR, *E*_ph_ = 20 keV, *W*_±_ = 50 eV, and find *J_d_* ≪ 60 pA mm^−2^. There is one further complication. The fluctuations in the dark current are not actually due to shot noise alone. There is a 1/*f* contribution that can be quite significant and more than the shot noise [115]. Since 1/*f* spectral power density scales with *I_d_*^2^, the current should be even lower than the rough estimate above. Suppose that we can express the spectral power density of the 1/*f* noise as $S=C_{\text{flicker}}I_{d}^{2}/f$ where *C*_flicker_ is a constant that characterizes the magnitude of 1/*f* fluctuation phenomena in the photoconductor material of interest. In the presence of double integration and subtraction (double sampling type of correction by subtracting the background charge), the variance of the charge collected is [115]

(13) $$\bar{\Delta Q^{2}}=\text{variance}=8\text{ln}(2)C_{\text{flicker}}I_{d}^{2}T^{2}$$

The carrier fluctuations in Equation (13) must be smaller than (Φ_ph_ *A*)^1/2^ (*α*_en_ / *α*) *E*_ph_ / *W*_±_. The constant *C_flicker_* can only be determined through 1/*f* experiments and often is not available for the photoconductor of interest. Indeed, many papers in the literature simply ignore the contribution of 1/*f* noise to the SNR. In the case of a-Se, some 1/*f* noise measurements have been reported and 1/*f* noise has been shown to be more dominant than shot noise over the frequencies of interest. The variance can be up to one hundred times larger, which puts the tolerable dark current requirement at around 1 pA mm^−2^.

**Figure 12.**
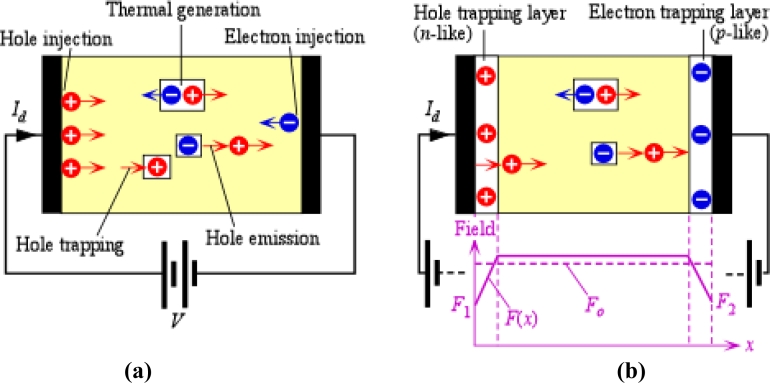
**(a)** In a single layer of a-Se sandwiched between two electrodes, the dark current is due to the injection of holes (most dominant) and electrons from the positive and negative contacts respectively as well as some thermal generation of electron and hole pairs or hole emission from defect states. **(b)** In the case of an x-ray photoconductor, there are two thin layers between the a-Se and the electrodes. The hole-trapping layer traps holes and allows electron transport (an *n*-like layer) and the electron-trapping layer traps electrons and allows hole transport (a *p*-like layer). The structure is often referred to as an *nip* type a-Se photoconductor. The radiation receiving side is the positive electrode.

The dark current in the case of practical a-Se based x-ray photoconductors can be reduced to an innocuous level by using thin blocking layers between the a-Se and the electrodes. Figure 12(a) shows a single layer sandwiched between two electrodes and identifies the sources of the dark current as the injection of holes and electrons from the positive and negative contacts respectively, possible thermal generation of electron and hole pairs or hole emission from defect states. In the case of a practical low-dark current x-ray photoconductor, there are two thin layers between the a-Se and the electrodes as shown in Figure 12(b); a “thin” layer refers to the fact that the blocking layer thickness is much smaller than the photoconductor thickness. The hole-trapping layer traps holes and allows electron transport (it is an *n*-like layer) and the electron-trapping layer traps electrons and allows hole transport (it is a *p*-like layer). The structure is often referred to as an *nip* type a-Se photoconductor since the a-Se layer can transport both holes and electrons, resembling an “intrinsic” semiconductor where both carriers play comparable roles. Such terminology is useful and convenient but must be used with care since the Fermi level in a-Se is near the center of the bandgap, and cannot be easily shifted by doping as in crystalline semiconductors. The required properties are obtained by changing the deep trap concentrations by suitably alloying and doping a-Se with the result that either injected holes or injected electrons are deeply trapped [80,116,117].

In order to prevent charge injection into the photoconductor, a blocking layer is used between the photoconductor and the electrode. The blocking layer has material properties that cause the trapping of carriers injected from the contact but allow the opposite sign carrier to be transported. Holes injected from the positive contact become trapped in the *n-*like layer and electrons injected from the negative contact become trapped in the *p*-like layer. The space charge is built-up within the *n*- and *p*- layers and modifies the field as shown in Figure 12(b). The actual fields, *F*_1_ and *F*_2_, at the positive and negative metal contacts now are lower than the corresponding values in the single layer, and hence the rates of hole and electron injection are significantly diminished. The dark current in such multi-layer a-Se photoconductors is about 3 orders of magnitude smaller than that in a single a-Se layer with the same thickness and applied field. In fact, by using *nip* structures, dark currents less than 1 pA mm^−2^ are routinely obtained for a-Se based photoconductors used in practical x-ray detector applications [15]. It is also possible to reduce the dark current in a-Se photoconductors by a similar amount by modifying the fabrication process itself. First, a thin film of stabilized a-Se is deposited onto a cold substrate to obtain the *n*-like layer in which only electrons can drift. This is annealed above the glass-transition temperature (*T_g_*) and then the thick *i*-layer is deposited on top of the *n*-layer during which the substrate is heated above *T_g_* [118,119].

There are two possible sources for the dark current in *nip* type a-Se photoconductor. First is the rate of actual injection of carriers from the contacts, which would have been much reduced with respect to the injection rate in single layer photoconductors but not zero (the signal current in fact weakens the blocking contact which requires some leakage to keep the contact in equilibrium). The second is the thermal generation in the bulk of the a-Se layer. The thermal generation process is likely to involve a mid-center defect state from which one can emit an electron and hole pair or simply emit only one type of carrier, most likely a hole. The emission of a hole would leave behind an immobile negatively charged center. The latter process controls the decay of the surface electrostatic potential in various a-Se alloy based photoreceptors [120]. Kabir and coworkers at Concordia University [121] have recently modeled the dark current in multilayer a-Se photoconductors by considering the following. The rates of hole and electron injection from the contacts (at metal/a-Se interface) are limited by a Schottky emission rate over some potential barrier for each type of carrier. Trapped holes in the *n*-like layer and electrons in the *p*-like layer modify the fields and hence control *F*_1_ and *F*_2_ in Figure 12(b). As *F*_1_ and *F*_2_ decrease, so does the dark current, and eventually a steady state is reached. Good agreement has been obtained with experimental results on practical detectors. The model neglects the contribution of thermal generation in the photoconductor, which should also be considered in future modeling, especially in thick a-Se photoconductors.

**Figure 13.**
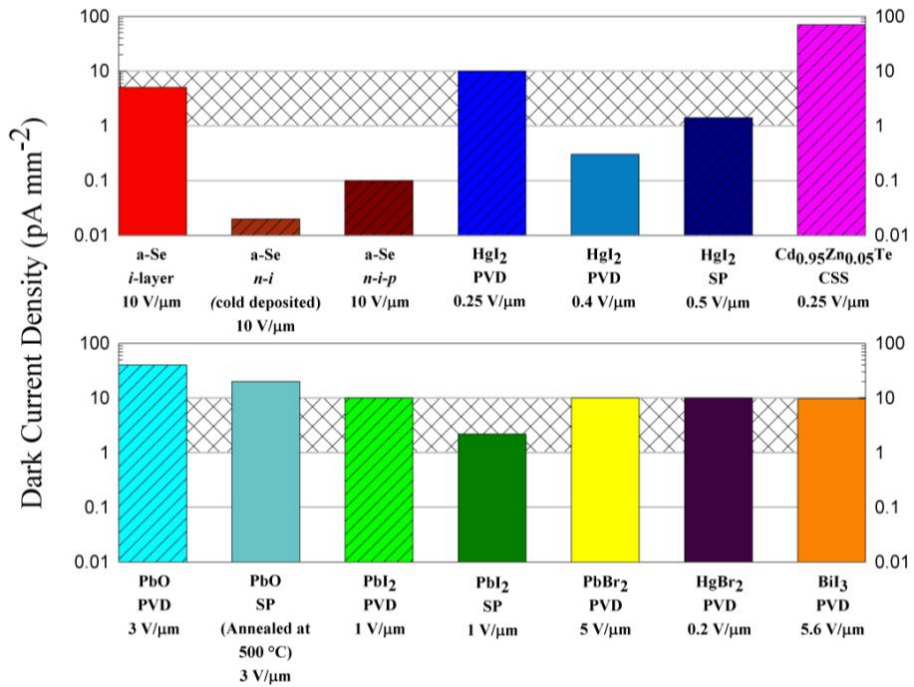
The best reported values to date of dark current density for a-Se and polycrystalline photoconductive layers. Note that most of these are measured at relatively low applied electric fields where it is questionable that the charge collection efficiency is adequate. It is not possible to scale these to the same field as the field dependence of the dark current is rarely linear and in general is unknown. All polycrystalline layers are labeled as deposited by physical vapour deposition (PVD), screen printing (SP) or close space sublimation (CSS). Solid colors represent values obtained from films that have not yet been used to obtain x-ray images, hashed bars represent values from demonstrated x-ray imagers. The grey hashed area represents the acceptable range for dark current in an FPXI. Data have been taken from various sources, including the following: a-Se (*i*-layer and *n-i-p*) from [15], a-Se (*n-i*) from [118], HgI_2_ (PVD at 0.25 V/μm and SP) from [51], HgI_2_ (PVD at 0.4 V/μm) from [122], PbI_2_ (PVD) from [56], PbI_2_ (SP) from [48], Cd_0.95_Zn_0.05_Te from [58], PbO (PVD) from [59], PbO (SP) from [123], PbBr_2_ and HgBr_2_ from [124] and BiI_3_ from [125].

Figure 13 compares the dark currents in a-Se and various other polycrystalline photoconductors, some of which have not yet been demonstrated in a prototype imager. The grey shaded region represents the 1–10 pA mm^−2^ range based on the maximum allowed dark current discussion above. What is notable is that the dark current in multilayer a-Se photoconductors is quite small compared to competing polycrystalline semiconductors. There are very few polycrystalline materials in which the dark current is below the acceptable value for an imaging sensor. We have also indicated the applied field for the reported dark current. The fields are not particularly large (e.g., 0.25–1 V μm^−1^) and at such low fields with the typical *μτ* ranges listed in Table 1, the CCE for some of the listed polycrystalline photoconductors is less than satisfactory [56]. It is not possible to simply scale the dark current to the same field in Figure 13 inasmuch as the field dependence of the dark current is rarely linear and in many cases it is unknown. While CCE would be near unity for the highest quality PVD HgI_2_ layer that has excellent *μτ* ranges for both electrons and holes (10^−3^ and 10^−5^ cm^2^ V^−1^ respectively), the same is not true for the screen printed HgI_2_ layers;see Table 1 for the *μτ* ranges. The dark current problem in polycrystalline photoconductors has not been fully solved in the sense that one can deposit the layer on the TFT-AMA, apply a sufficiently large electric field, maintain a low dark current, and achieve good CCE.

## 5. X-Ray Photoconductors with Avalanche Gain and Imaging Applications

Recently Karim and Rowlands proposed a large area digital x-ray imager that utilizes avalanche multiplication in a-Se [126]. To date, a-Se remains the only amorphous semiconductor in which there is clear evidence that the primary charge carriers (holes) can acquire enough energy from the applied field to initiate impact ionization and secondary charge creation [127–129]. Impact ionization at high fields results in avalanche multiplication, *M*, which depends exponentially on the photoconductor layer thickness [130,131]. Experiments on hole impact ionization in a-Se indicate that avalanche is initiated at electric fields exceeding a certain avalanche multiplication threshold, *F*_th_. The latter is about 70 V μm^−1^ for a-Se layers thicker than 15 μm; *F*_th_ depends slightly on the a-Se thickness. Thus far, a maximum avalanche gain of 10^3^ has been demonstrated for a 30 μm thick a-Se layer at a field of 92 V μm^−1^ [127]. The avalanche gain capability of a-Se photoconductors potentially provides practical solutions to a number of important applications in the field of medical image detectors, inasmuch as it promises to increase a-Se’s x-ray to charge conversion efficiency and lead to a-Se detectors that are effectively quantum noise limited in operation at all exposure levels. A further often overlooked advantage of avalanche multiplication is to increase the dynamic range of a system by permitting the maximum signal capacity to be adjusted by changing the effective multiplication gain. There have been a number of recent studies by Rowlands and coworkers that have examined the use of avalanche in a-Se for medical imaging applications [132–137] as well as in protein crystallography [138,139]. These imaging applications use both indirect and direct x-ray detection techniques and then avalanche, or carrier multiplication, in a-Se to achieve gain. Large area a-Se based direct conversion FPXIs with avalanche gain have not yet been demonstrated. Small area x-ray imaging using an a-Se Harpicon has been demonstrated and shows the potential of avalanche gain in rendering the detector quantum noise limited. A feasibility case study of a large area FPXI with avalanche gain has been recently undertaken by Wronski and Rowlands [140]. The latter study concludes that an a-Se flat-panel imager structure with avalanche gain enables high-resolution fully quantum noise limited x-ray imaging over a wide exposure range.

**Figure 14.**
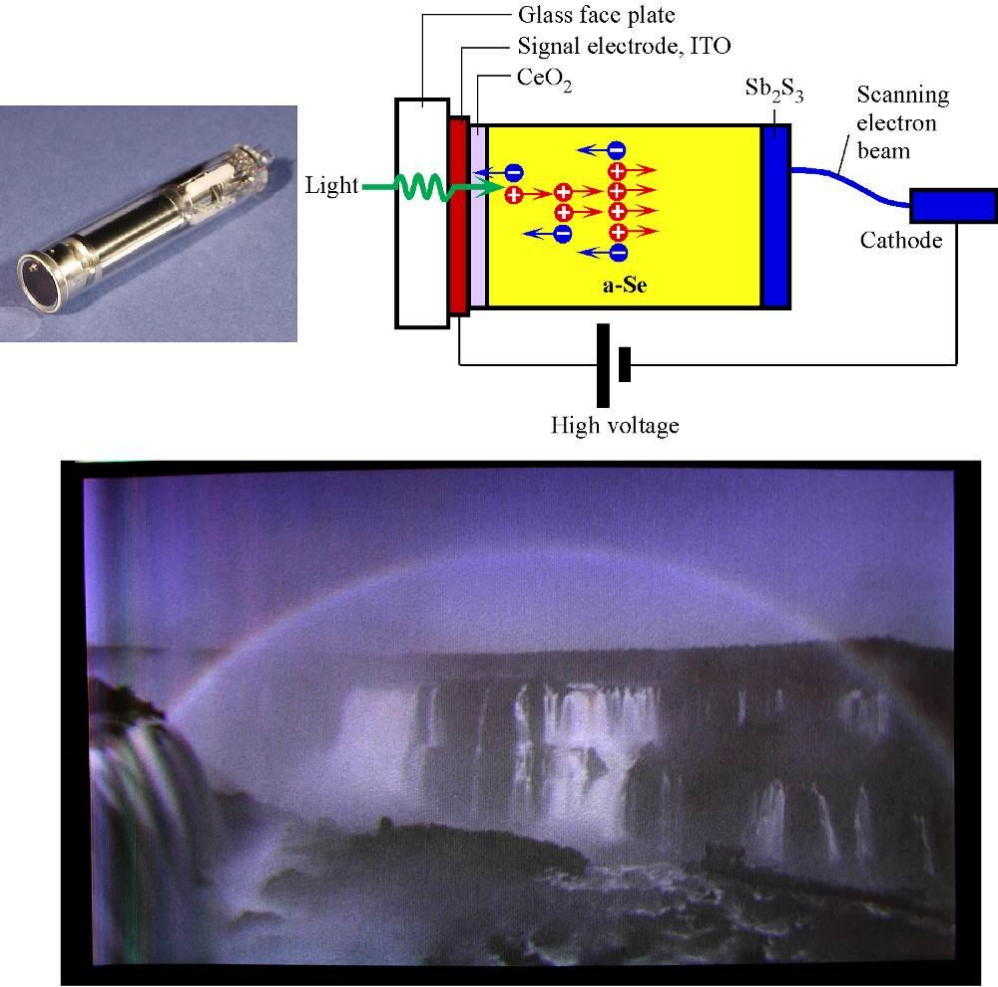
Top left. A HARP tube is a TV pick-up (video) tube with avalanche gain; it is called a Harpicon. Top right. A schematic illustration of the HARP and its operation under avalanche. Bottom. A snap-shot image from a real time movie of a rainbow formed under moonlight at night at Iguazu Falls, Brazil, taken by a HDTV-Harpicon. (Courtesy of Dr. Kenkichi Tanioka, NHK, Japan).

The application of a high electric field to an a-Se photoconductor that would generate avalanche multiplication required the development of a special multilayer photoconductor structure; the goal was to use these structures in TV video tubes [141]. The a-Se based photoconductive target with avalanche gain is called a HARP, an acronym for High-gain Avalanche Rushing Photoconductor structure. The photoconductive a-Se layer is confined between specially designed blocking layers which almost completely block charge injection at high fields [142]: a thin layer of CeO_2_ (∼20 nm) on the front side (light receiving side) of the a-Se layer and Sb_2_S_3_ (∼500 nm) on the back side, which receives the electron beam. Figure 14 shows the typical structure of a HARP. The CeO_2_ and Sb_2_S_3_ layers serve as blocking layers for holes and electrons, respectively. The blocking mechanisms in these two layers are different from each other. CeO_2_ is an *n*-type wide bandgap material (*E_g_* of 3.4 eV, *E_F_* about 0.5 eV below *E_c_*) and prevents the injection of holes from the anode by forming a high potential barrier to holes; electrons can drift freely through the CeO_2_/a-Se interface. The Sb_2_S_3_ layer on the other hand has a bandgap slightly narrower than that of a-Se, but it contains a large number of deep electron traps which, when filled, form a negative space-charge barrier, thus stopping the injection of electrons from the cathode; at the same time holes can flow freely through the a-Se-/Sb_2_S_3_ interface. The HARP target was designed to be used in a vacuum device, that is, in the TV video tube (a TV pick-up tube). These tubes have been called *Harpicons*.

a-Se HARP structures have been developed by NHK in Japan as photoconductive targets of broadcast video cameras and are now used routinely for electronic news gathering in HDTV, *i.e.*, operation at low light conditions [143]. For use in optical imaging, a-Se HARP structure is deposited on a glass substrate covered with an ITO (indium tin oxide) coating, which serves as a transparent anode. The back of the a-Se HARP structure is free, that is, it has no physical electrode, which allows it to form a latent charge image. A scanning electron beam serves as a virtual cathode, biasing the free surface (see Figure 14). Light photons incident on the front a-Se surface through a positively biased ITO electrode are absorbed and create EHPs. The freed holes drift to the free surface of the a-Se layer and if the electric field exceeds *F*_th_, the drifting holes undergo avalanche multiplication. The holes accumulate as a latent charge image at the free surface in an amount proportional to the incident light intensity. An electron beam scans the free surface, completing the circuit, and enabling the accumulated positive charge to be sensed by the ITO electrode as a current. There have been many examples on the uses of Harpicons in low-light level applications in which they outperform all standard imaging chips; Figure 14 has a sample image from a real-time movie of a rainbow observed under moonlight at night.

**Figure 15.**
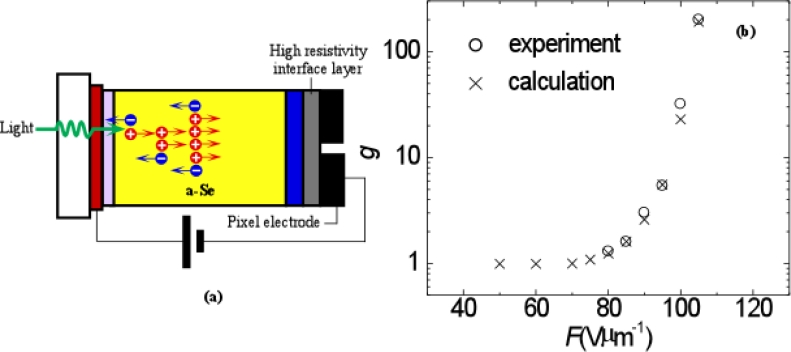
**(a)** A modified-HARP structure with an RIL (resistive interface layer) and the development of a fully electroded image sensor, which can be used in direct and indirect conversion detectors. **(b)** Experimentally measured field dependence of avalanche gain for 15 μm thick modified-HARP structure (open circles) in comparison with the theoretical field dependence of the avalanche gain (crosses) for the same layer thickness.

Although the electron beam readout is compatible with HDTV, its use in digital medical imaging is cumbersome, and the electron beam should be replaced by a two-dimensional array of metal pixels. Unfortunately, if metal electrodes are deposited directly on a HARP device, the dielectric breakdown occurs at the contact edges due to the local enhancement of the electric field. An incipient breakdown causes a high current flow that can induce irreversible damage of an area adjacent to the contact because of Joule heating. This problem can be overcome with a *modified*-HARP structure where a thin resistive interface layer (RIL) is introduced between the avalanche a-Se structure and the pixel electrodes as shown in Figure 15(a).

We have recently shown that modified HARP structure with 15 μm thick a-Se layer and ∼1 μm RIL made of a semi-insulating polymer—cellulose acetate (CA), can reliably withstand an electric field of 105 V μm^−1^ [144]. A high electric field could be cycled up and down many times with no noticeable change in the properties. Figure 15(b) shows the experimentally measured field dependence of the avalanche multiplication gain, *g*, for a 15 μm thick modified-HARP structure. As it can be seen from Figure 15(b), *g* = 200 is reached at 105 V μm^−1^, which is the maximum theoretical gain for this thickness of a-Se layer [145]. Furthermore, experimental results agree well with theoretical gain values calculated using well-known impact ionization coefficients for holes [131].

**Figure 16.**
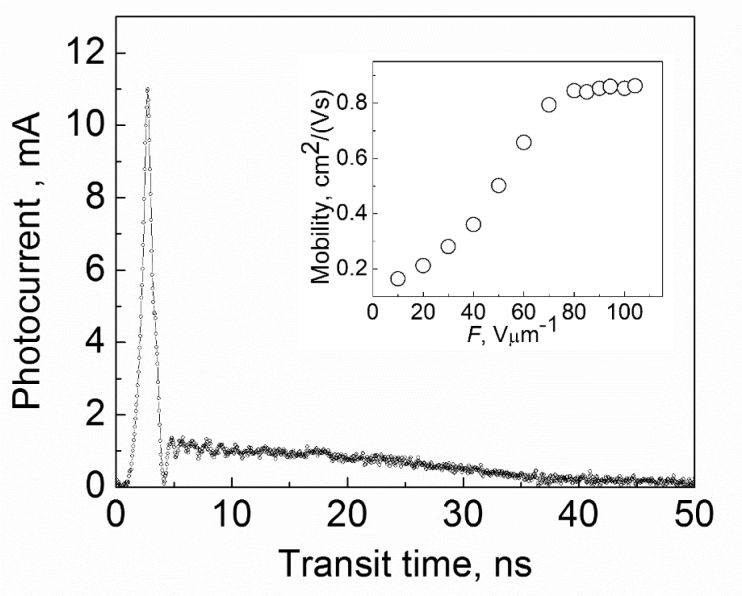
TOF signal from a-Se HARP blocking structure with a RIL in avalanche regime for *F* = 100 V μm^−1^. The inset shows the dependence of the drift mobility on the applied field.

Time-of-Flight (TOF) transient photoconductivity measurements serve as a very useful tool in studying the transport and multiplication of charge carriers in the modified-HARP device [146]. Figure 16 shows a typical TOF photocurrent pulse measured at a field higher than the threshold field for avalanche. The TOF photocurrent evinces an initial sharp rise, due to the motion of avalanching holes, and a comparatively long tail, due to the slow motion of secondary nonavalanching electrons created in the bulk of the a-Se as a result of impact ionization [147]; a typical TOF photocurrent in the avalanche regime is shown in Figure 16. Theoretical calculations of the TOF signal based on the motion of both electrons and holes, but allowing only the holes to avalanche, match the observed TOF photocurrents, and provide insight into the operation of the modified-HARP. We have been able to extract the field dependence of the hole drift mobility by matching the theoretical calculations with TOF photocurrents, which is shown in the inset of Figure 16.

It should be noted that both the shape of the photocurrent and the field dependence of the hole mobility are identical to those obtained in “prototype” a-Se samples by Juska and Arlauskas [130,147], who discovered avalanche multiplication in a-Se in the early 80’s using metal/polymer/a-Se/polymer/metal sandwich structures that have thin polyethyleneteraphalate *insulating* layers between the metal electrodes and a-Se. Such structures permitted the application of avalanche fields without charge injection from the contacts, but unfortunately did not allow for the charge to fully exit the a-Se/polymer structure. Thus, a-Se insulating structures were not practical, though they served to demonstrate the existence of avalanche multiplication in this material. In the recent work carried out at Lakehead University and the Thuder Bay Regional Research Institute, we were able to show that both the modified (with the RIL) and the original HARP devices exhibit almost identical charge transport, which means that that RIL does not degrade a-Se transport properties while enabling its stable operation in the avalanche regime without a sudden full breakdown. Modified a-Se HARP structures represent the future of *a*-Se photodetectors in medical x-ray imaging in both direct conversion detectors for low energy applications; and in indirect conversion detectors for fluoroscopic applications [131,137]. Another important potential application for avalanche a-Se photoconductors is in protein crystallography, which involves measuring the intensities in the diffraction pattern, and needs a sensitive detector with a large dynamic range, as recently discussed in [138].

A final note on the observation of avalanche gain in amorphous semiconductors is appropriate. Amorphous semiconductors have low carrier mobilities because of the random potential fluctuations in their structure. It was therefore quite surprising that avalanche multiplication was actually observed in a-Se, and it created some controversy at the time. It turns out that impact ionization in a-Se can be readily explained by invoking the lucky drift model in which carriers can become scattered by potential fluctuations and can still gain sufficient energy at high fields to cause impact ionization [127,148].

## 6. Active Pixel Sensor Based X-Ray Imagers

As mentioned in Section 1, passive pixel sensors (PPS) represent the default pixel configuration in active matrix flat-panel imagers for X-ray imaging applications [1,149]. While the PPS circuit has the advantage of being compact and amenable to high-resolution imaging, small PPS output signals, under conditions of low exposures and/or high spatial resolution, are swamped by an external column charge amplifier (CA) and data line thermal noise. Active pixel sensor (APS) circuits are improvements over PPS circuits, primarily due to an increased pixel signal-to-noise ratio (SNR) [35,150]. The polycrystalline silicon (poly-Si) thin film technology offers the ability to fabricate TFTs with higher gain due to the higher drift mobility of electrons in poly-Si thin films vis-à-vis the a-Si:H technology. Thus, the poly-Si technology has the ability to implement APS circuits for medical X-ray imaging, which represents an attractive way to increase the sensitivity at the pixel level, as reported previously [34]. However, the large-area poly-Si technology carries the additional constraints of limited availability, with only a few foundries that are capable of manufacturing poly-Si devices, and lower uniformity over large areas (e.g., 30 cm × 40 cm) with respect to a-Si:H technology. In addition, poly-Si TFTs tend to be more noisy than a-Si:H TFTs in terms of their low-frequency noise performance [151]; the excess noise depends on the passivation of the grain boundaries.

Unlike CMOS APS imagers, which operate on a voltage transfer principle [152], a-Si:H APS imagers need to make use of the current transfer operation [35] due to the very long readout times (>100 μs) associated with the voltage-transfer operation in a-Si:H technology. The speed constraints for voltage-transfer a-Si pixels stem from two factors: (a) low a-Si:H thin-film transistor (TFT) carrier mobility (∼0.5 cm^2^/Vs) and (b) large imaging array column capacitances (40–100 pF) [35]. Long readout times pose a problem in the case of large-area digital X-ray imaging modalities requiring higher frame rates, such as real-time fluoroscopy or 3-D mammography tomosynthesis. Furthermore, it is precisely at high frame rates where the SNR decreases due to the low X-ray doses required for patient safety.

**Figure 17.**
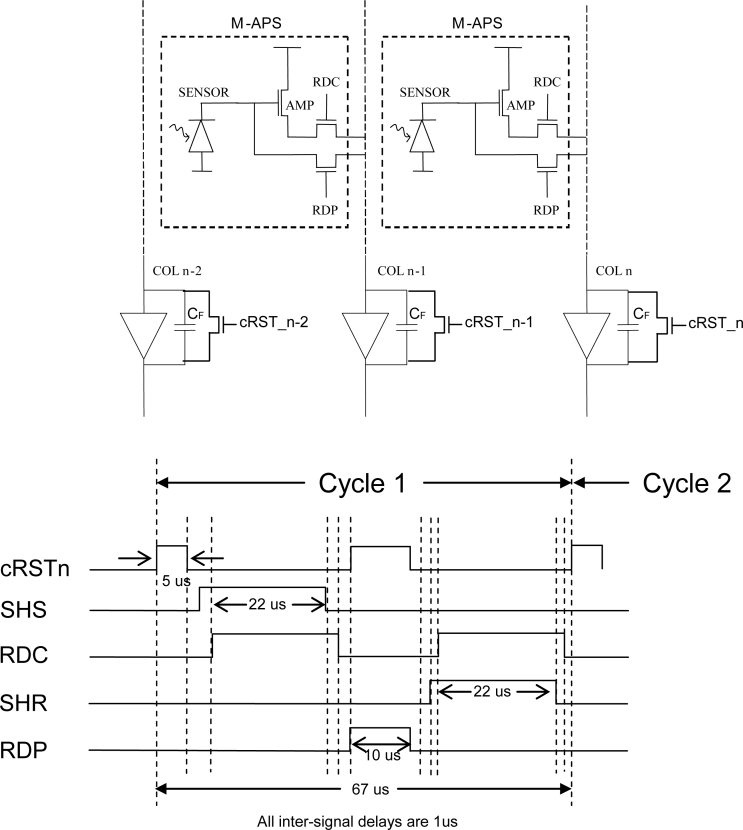
Three transistor APS pixel circuit and the timing diagram.

Operating in the current transfer mode while solving the problem of long readout times imposes a linearity constraint on the imaging pixel, which is violated by large voltage swings at the sense node. In order to circumvent this nonlinearity at the sense node, a four-transistor (4T) multimode APS and PPS pixel was first employed in [153], where each pixel is effectively read out in both the APS and PPS modes. Note that all three transistors, shown in Figure 17, and two-transistor multimode pixel designs (which are variants of the 4T design) have also been reported previously at the pixel level [154,155].

**Figure 18.**
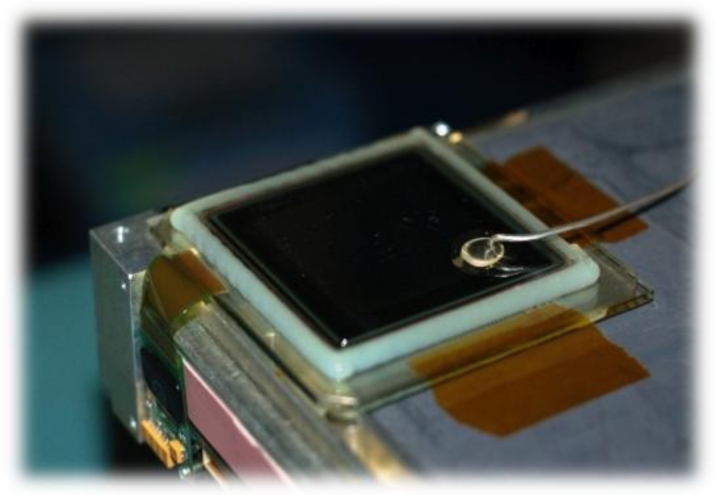
A direct conversion sensor based on coating a 64 × 64 pixel array with a-Se.

**Figure 19.**
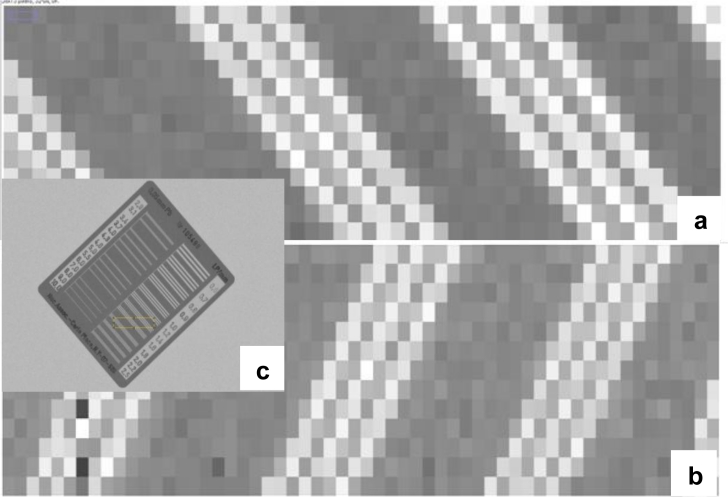
X-ray resolution image test from **(a)** a commercial FPD14 PPS array at 1.6 lp/mm shown in center of image **(b)** prototype 64 × 64 APS array at 2.0 lp/mm shown in center of image and **(c)** the resolution target.

Recently, a proof-of-concept 64 × 64 4T APS array for medical X-ray imaging was fabricated using the a-Si:H technology and coated with an amorphous selenium photoconductor to produce an X-ray sensor [156]. The low-exposure measurements (in the microroentgen range) including X-ray image results obtained using an in-house fabricated 4T a-Si APS array demonstrated, for the first time, that signals as low as 1.5 μR were measurable in an a-Si APS array. A 4T pixel design [153,156] was chosen in order to utilize the same external off-panel readout electronics used by a commercial PPS imager made by Anrad Corporation. Figure 18 shows the in-house prototype device sitting in a test jig while Figure 19 shows images taken with the prototype device compared to a commercial PPS imager device.

## Figures and Tables

**Table 1.** Some typical or expected properties of selected x-ray photoconductors for large area applications. *δ* is the attenuation depth at the shown photon energy, either 20 keV or 60 keV. From various references and combined selectively. *μτ* represents the carrier range. From various references including [14,16,67] and those listed in the table. (SP refers to screen printing, and PVD to physical vapor deposition).

Photoconductor State Preparation	*δ* at 20 keV *δ* at 60 keV	*E_g_* eV	*W*_±_ eV	Electron *μ_e_τ_e_* (cm^2^/V)	Hole *μ_h_τ_h_* (cm^2^/V)
Stabilized a-Se Amorphous Vacuum deposition [15]	49 μm 998 μm	2.2	45 at 10 V/μm 20 at 30 V/μm	3×10^−7^–10^−5^	10^−6^–6 × 10^−5^
HgI_2_ Polycrystalline PVD [68]	32 μm 252 μm	2.1	5	10^−5^–10^−3^	10^−6^–10^−5^
HgI_2_ Polycrystalline SP [56,68–70]	32 μm 252 μm	2.1	5	10^−6^–10^−5^	∼10^−7^
Cd_.95_Zn_.05_Te Polycrystalline Vacuum deposition (sublimination)	80 μm 250 μm	1.7	5	∼2 × 10^−4^	∼3 × 10^−6^
PbI_2_, Polycrystalline Normally PVD [48,49]	28 μm 259 μm	2.3	5	7 × 10^−8^	∼2 × 10^−6^ [71]
PbO, Polycrystalline Vacuum deposition	12 μm 218 μm	1.9	8–20	5 × 10^−7^	small
TlBr Polycrystalline Vacuum deposition [46]	18 μm 317 μm	2.7	6.5	small	1.5–3 × 10^−6^

**Table 2.** Effects of charge carrier trapping in the x-ray photoconductor layer (e.g., a-Se) on various image sensor metrics. We assume that the radiation receiving electrode is positively biased and the negative electrode is pixellated. Reversal of the biasing potential results in identical effects for the opposite charge.

Phenomenon	Primary observable effects	Comment	Example reference
Hole or electron trapping. The capture of carriers into deep traps.	Reduction in sensitivity Reduction in DQE(0) Reduction in MTF for hole trapping Increase in MTF at high spatial frequencies for electron trapping	Radiation receiving electrode is positive	[82,86–88, 90,91]
Hole recombination with previously trapped electrons	Reduction in sensitivity Ghosting	Similar effects if electrons recombine with previously trapped holes	[81]
Bulk electron and hole recombination	Reduction in sensitivity Loss of linearity in collected charge *vs.* exposure	At very high doses that lead to large concentrations of drifting electrons and holes in the bulk.	[95]

## Acronyms

4T: Four Transistor
AMA: Active Matrix Array
APS: Active Pixel Sensor
a-Se: Amorphous Selenium
CA: Charge Amplifier
CCE: Charge Collection Efficiency
CMOS: Complimentary Metal-Oxide-Semiconductor
CSS: Close Space Sublimation
DQE: Detective Quantum Efficiency
ECCE: Electron Charge Collection Efficiency
EHP: Electron-Hole Pair
FF: Fill Factor
FPXI: Flat Panel X-ray Imager
HARP: High-gain Avalanche Rushing Photoconductor structure
HCCE: Hole Charge Collection Efficiency
HDTV: High Definition Television
IFTOF: Interrupted Field Time-Of-Flight
ITO: Indium-Tin-Oxide
MTF: Modulation Transfer Function
NPS: Noise Power Spectrum
PPS: Passive Pixel Sensor
PVD: Physical Vapor Deposition
QE: Quantum Efficiency
R: Roentgen
RIL: Resistive Interface Layer
RMS: Root Mean Square
SNR: Signal to Noise Ratio
SP: Screen Printing
TFT: Thin Film Transistor
TOF: Time-of-Flight
TV: Television

## Symbols

*a*: Pixel size (μm)
*A*: Area (cm^2^)
*A_Q_*: Quantum efficiency
*C*_flicker_: Constant in the 1/*f* (flicker) noise power spectral density equation
*D*: Diffusion coefficient (cm^2^ s^−1^)
*e*: Elementary charge
*E*_binding_: Electron binding energy (eV)
*E_g_*: Bandgap (eV)
*E*_ph_: Photon energy (eV)
*F*: Electric field (V cm^−1^ or V μm^−1^)
*f*: Spatial frequency (cycles mm^−1^)
*f_N_*: Nyquist frequency (cycles mm^−1^)
*F*_th_: Avalanche multiplication threshold Field (V μm^−1^)
*g*: Gain
*g*: Avalanche multiplication gain
*G*: Detector gain
*I_d_*: Dark current (pA)
*J_d_*: Dark current density (pA mm^−2^)
*k*: Boltzmann constant
*L*: Photoconductor thickness (μm)
*M*: Avalanche multiplication factor
*N_d_*: Number of charge carriers due to dark current
*Q*: Electric charge (C)
*q*_0_: Incident quanta
*S_x_*: X-ray Sensitivity
*S_N_*: Noise power spectrum
*T*: Temperature (K)
*T_g_*: Glass transition temperature (K)
*t_t_*: Transit time (s)
*V*_th_: Thermal voltage (V)
*W_±_*: Electron-hole pair creation energy (eV)
*X*: Exposure (R)
*x_e(h)_*: Schubweg per unit thickness for electrons(holes)
*Z*: Atomic number
*α*: Linear attenuation coefficient (m^−1^ or μm^−1^)
*α*_en_: Energy absorption coefficient (m^−1^ or μm^−1^)
*δ*: Attenuation depth (m or μm)
Δ: Normalized attenuation depth, *δ/L*
Δ*y*_diffusion_: Root mean square diffusion distance (m)
*η*_CC_: Charge collection efficiency
*η*_ECC_: Electron charge collection efficiency
*η*_HCC_: Hole charge collection efficiency
*μ*: Drift mobility (cm^2^ V^−1^ s^−1^)
*μτ*: Carrier range (cm^2^ V^−1^)
*μτF*: Schubweg (mm)
*ρ*: Density (g cm^−3^)
*σ^2^*: Variance
*τ*: Mean lifetime, mean deep trapping time (s or *μ*s)
Φ: Photon fluence (photons cm^−2^)
